# A Network-guided Association Mapping Approach from DNA Methylation to Disease

**DOI:** 10.1038/s41598-019-42010-6

**Published:** 2019-04-03

**Authors:** Lin Yuan, De-Shuang Huang

**Affiliations:** Institute of Machine Learning and Systems Biology, College of Electronic and Information Engineering, Tongji University, Shanghai, 201804 P.R. China

## Abstract

Aberrant DNA methylation may contribute to development of cancer. However, understanding the associations between DNA methylation and cancer remains a challenge because of the complex mechanisms involved in the associations and insufficient sample sizes. The unprecedented wealth of DNA methylation, gene expression and disease status data give us a new opportunity to design machine learning methods to investigate the underlying associated mechanisms. In this paper, we propose a network-guided association mapping approach from DNA methylation to disease (NAMDD). Compared with existing methods, NAMDD finds methylation-disease path associations by integrating analysis of multiple data combined with a stability selection strategy, thereby mining more information in the datasets and improving the quality of resultant methylation sites. The experimental results on both synthetic and real ovarian cancer data show that NAMDD substantially outperforms former disease-related methylation site research methods (including NsRRR and PCLOGIT) under false positive control. Furthermore, we applied NAMDD to ovarian cancer data, identified significant path associations and provided hypothetical biological path associations to explain our findings.

## Introduction

Epigenetics is defined as heritable changes in gene expression that are not due to any alteration in the DNA sequence^1^. The importance of epigenetics lies in offering a partial explanation of phenomena that classic genetics alone cannot explain^2^ and has thereby attracted increasingly more attention. DNA methylation, one of the best-known epigenetic markers, plays a critical role in modifying gene expression. Additionally, emerging results indicate that aberrant DNA methylation contributes to the development of cancers^3,4^.

One of the fundamental problems associated with studying DNA methylation is understanding how aberrant DNA methylation affects tumorigenesis^5^. In recent decades, many works have been proposed to detect the associations between DNA methylation and complex diseases (e.g., ovarian cancer and colorectal cancer). Statistical classification-based methods were used to develop innovative solutions to identify disease-related methylation sites. For example, Houseman *et al*.^6^ proposed a model-based recursive-partitioning algorithm to select disease-related DNA methylation site clusters. Kuan *et al*.^7^ developed a statistical framework based on a weighted model to identify informative CpG loci. However, recent studies have demonstrated that aberrant DNA methylation sites generally only affect a small proportion of genes^8^; therefore, the input data are generally sparse or group sparse. Computationally, these statistical classification-based methods normally ignore the sparsity of input data, which may affect the quality of predicted results. Following these statistical classification-based methods, a penalized conditional logistic regression model with L1 penalty and squared L2 penalty (PCLOGIT)^9^ was proposed to focus on finding effective predictors in the case of sparsity and group sparsity among methylation data. This method outperforms the statistical classification-based methods and traditional variable selection methods, such as fused LASSO^10^ and the elastic-net (Enet) procedure^11^. However, the relationship between DNA methylation sites and complex diseases is still unclear due to complex mechanisms. Meanwhile, compared with the convex optimization problem involving the L1 and L2 penalty functions, many results show that improved performance can be obtained by using nonconvex optimization^12,13^.

Recently, many researchers have been working on investigating the relationship between DNA methylation sites and gene traits to bridge the gap between methylation sites and complex diseases. For example, Pearson’s correlation coefficient^14^, the maximal information coefficient^15^ and standard two-sample univariate statistical tests (e.g., *t*-test)^16^ have been used to find methylation-gene associations. These methods focus on revealing associated information between a DNA methylation site and a gene. However, more than one aberrant DNA methylation site can affect gene expression values. Network-sparse reduced-rank regression (NsRRR)^17^ was proposed to tackle this limitation; it is a multivariate regression model for the simultaneous selection of highly predictive DNA methylation predictors and the most predictable gene expression profiles. This method outperforms existing methods. However, this method requires sufficient and accurate prior information, including the number of DNA methylation sites and genes to be predicted, an adjacency matrix for the DNA methylation sites and an adjacency matrix for the predictable genes. Unfortunately, it is difficult to provide sufficient and accurate prior information for the analysis of large-scale cancer data. Second, because there is no consideration of the relationship between genes and disease status, results obtained by this method may contain many false positives (i.e., a normal DNA methylation site is identified as a disease-related methylation site).

**Figure 1:**
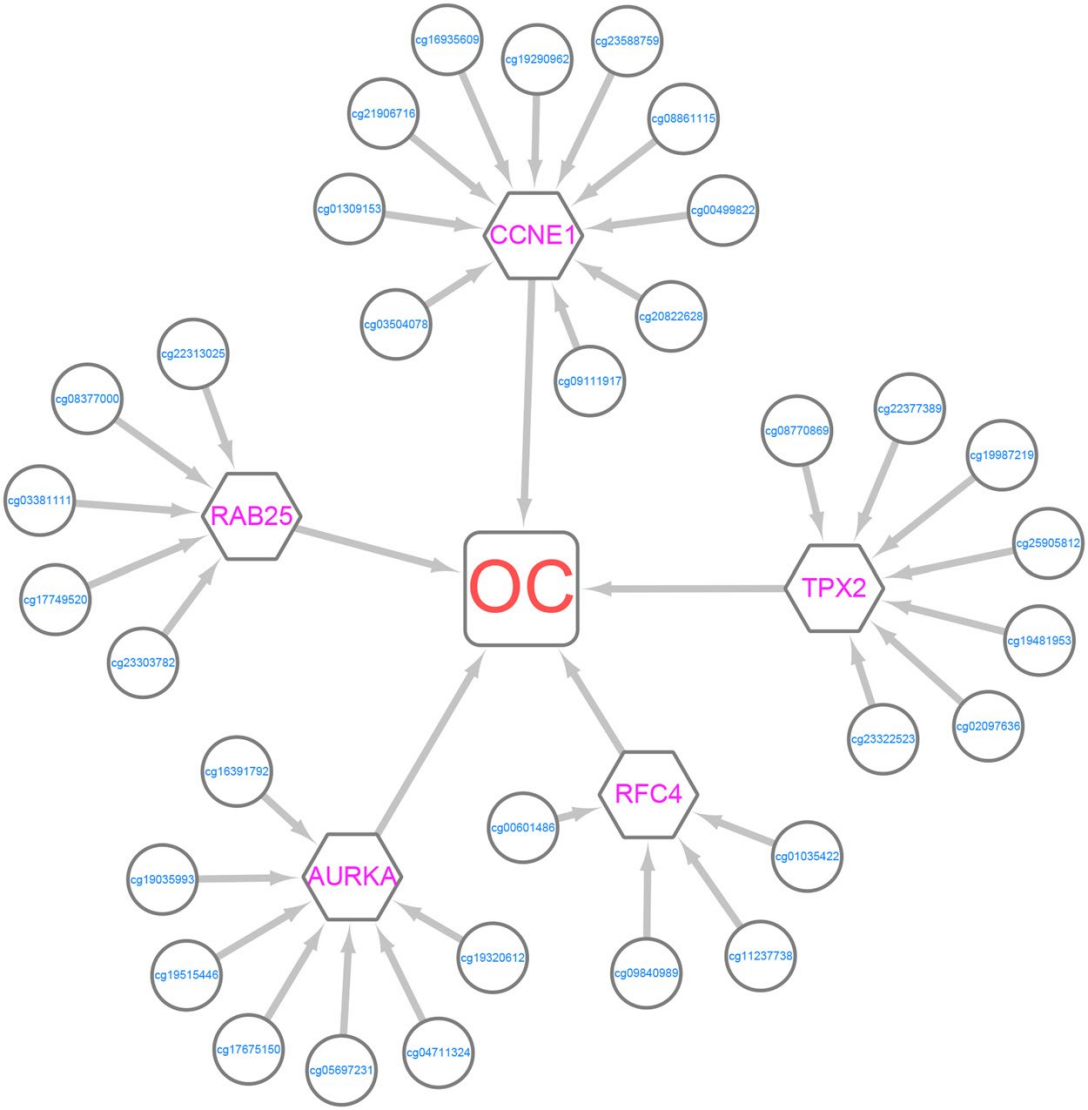
An association network for ovarian cancer identified by NAMDD. Circles represent DNA methylation sites, hexagons are genes, rounded corner quadrilateral is ovarian cancer, and edges represent association between nodes.

To tackle these limitations of disease-related methylation site research methods, we propose a path association analysis method (i.e., association analysis among DNA methylation sites, genes and disease), NAMDD (network-guided association mapping from DNA methylation to disease), to detect disease-related methylation sites by leveraging a hybrid dataset consisting of DNA methylation, gene expression and disease status data. NAMDD integrates three kinds of datasets to discover path associations from DNA methylation sites to disease using gene expression data. Let us first introduce the concept of path associations. Consider a network in which nodes represent DNA methylation sites, gene traits, or disease and edges are assigned scores and represent the relevance of a pair of nodes. DNA methylation sites, genes, and disease are connected to construct an association network. Path association means an association from a DNA methylation site to a gene and from the gene to disease. Figure 1 illustrates an association network identified by NAMDD using an ovarian cancer (OC) dataset. To construct an association network, we first adopt a nonconvex alternating direction method of multipliers (NcADMM)^12^ algorithm, which is an efficient algorithm for computing sparse and group-sparse representations in compressive sensing, to examine associations between DNA methylation sites and genes; meanwhile, L1-regularized logistic regression (LLR) is used to find disease-related genes. Both of these methods are used under stability selection^18^, which can effectively control false positives. Finally, based on the edge weight scores from the previous step, we use a path search algorithm to discover top *K* path associations and significant DNA methylation sites. To ensure the computational efficiency of NAMDD for real large-scale data, we propose a screening method to improve the efficiency of the algorithm.

Our proposed approach (NAMDD) has advantages over existing disease-related methylation site research methods. Initially, compared with single methylation site analysis, NAMDD can find weighted edges and consider all DNA methylation sites and genes simultaneously. Furthermore, NAMDD integrates three kinds of data (DNA methylation, gene expression and disease status data), which helps us to bridge the gap between methylation sites and disease (i.e., methylation sites affect gene traits and gene traits influence disease) and better understand methylation-cancer complex mechanisms. Finally, compared with methods that need prior information, NAMDD does not require any prior information; therefore, it is more suitable for large-scale datasets.

In our experiments, we first compared the receiver operating characteristic (ROC) performance of NAMDD with those of two well-known disease-related methylation site research methods (NsRRR and PCLOGIT) using eight kinds of synthetic datasets. The results show that NAMDD can take advantage of gene expression data to significantly improve the performance in detecting disease-related DNA methylation sites under false positive control. The boxplots show that the stability of NAMDD is better than those of NsRRR and PCLOGIT. NAMDD achieved a better performance on the ovarian cancer datasets from The Cancer Genome Atlas (TCGA). We then applied NAMDD to ovarian cancer datasets and identified 389 significant path associations, among which we analyzed the statistical significance of DNA methylation sites and genes from top 22 paths. The statistical significance of the top 22 paths were estimated using a permutation test. We also provided hypothetical biological path associations to explain our findings. The software of NAMDD is available at https://github.com/nathanyl/NAMDD.

## Methods

Before introducing our method, we summarize the notations used in this article. Matrices are denoted by boldface uppercase, vectors are denoted by boldface lowercase, and scalars are denoted by lowercase letters. We denote the DNA methylation predictor matrix by **X** ∈ **R**^*N*×*P*^, where *N* represents the number of samples and *P* represents the number of DNA methylation sites. **x**_*j*_ represents the *j*-th column of the DNA methylation predictor matrix, **x**^*i*^ represents the *i*-th row of the matrix, and ${x}_{j}^{i}$ represents the (*i*, *j*) matrix entry. Meanwhile, the gene expression matrix is denoted by **Y** ∈ **R**^*N*×*Q*^ with *N* samples and *Q* gene traits, and the disease status matrix is denoted by **Z** ∈ **R**^*N*×*K*^ with *K* diseases.

Next, we show how to discover disease-related path associations. We also describe how to find DNA methylation sites that affect gene traits, identify genes that affect disease, construct an association network, and define a path score formula that evaluates the significance of path associations. Additionally, based on the path scores, we use a path search algorithm to discover top *K* path associations and significant DNA methylation sites. For large-scale datasets, we also propose a screening method to improve the efficiency of the algorithm.

### Discovering paths in an association network

Given datasets containing DNA methylation, gene expression, and disease status data, we used DNA methylation sites, gene traits, and disease status as nodes in an association network. We describe how to construct edges between two nodes using a nonconvex alternating direction method of multipliers (NcADMM) algorithm for group sparsity with sparse groups^12^ and L1-regularized logistic regression (LLR)^10^ under stability selection^18^. NcADMM and LLR provide powerful techniques to discover associations between DNA methylation sites and gene traits or associations between gene traits and diseases, and the stability selection provides an effective way to control false positives.

The advantages of using NcADMM and LLR over single DNA methylation site analysis are reflected in two aspects. First, NcADMM and LLR are multivariate regression methods that can consider all DNA methylation sites or gene traits simultaneously. As a result, they can handle large-scale data. Second, NcADMM takes advantage of sparsity and group structure information from DNA methylation data, while LLR takes advantage of sparse mapping from gene traits to disease (i.e., a small number of genes are related to disease).

To detect informative edges between DNA methylation sites and genes, the original model is:

1 $$\mathop{{\rm{\min }}}\limits_{{\bf{B}}}\,\frac{1}{2}{\Vert {\bf{XB}}-{\bf{Y}}\Vert }_{F}^{2}+\alpha {\Vert {\bf{B}}\Vert }_{1}+\beta \sum _{i=1}^{P}{\Vert {{\bf{b}}}^{i}\Vert }_{2}$$

where **B** ∈ **R**^*P*×*Q*^ is a regression coefficient matrix whose nonzero entries represent associations between DNA methylation sites and genes, **X** is a DNA methylation predictor matrix and **Y** is a gene expression matrix. Here, ${\Vert \cdot \Vert }_{1}$ is the element-wise L1-norm, ${\Vert \cdot \Vert }_{2}$ is the L2-norm, ${\Vert \cdot \Vert }_{F}$ is the Frobenius (element-wise L2) norm and **b**^*i*^ is the *i*-th row of **B**. The second term ${\Vert {\bf{B}}\Vert }_{1}$ promotes the sparsity of the overall data, and the third term $\sum _{i=1}^{P}{\Vert {{\bf{b}}}^{i}\Vert }_{2}$ promotes group sparsity in the sense that only a few DNA methylation sites affect gene traits. We first use an alternating direction method of multipliers (ADMM) approach, which uses variable splitting, to decompose the original problem into easily solvable sub-problems^19^. The auxiliary variable **W** is used to split the data as follows:

2 $$\mathop{{\rm{\min }}}\limits_{{\bf{W}},{\bf{B}}}\frac{1}{2}{\Vert {\bf{XB}}-{\bf{Y}}\Vert }_{F}^{2}+\alpha {\Vert {\bf{W}}\Vert }_{1}+\beta \sum _{i=1}^{P}{\Vert {{\bf{w}}}^{i}\Vert }_{2}+\frac{1}{2}{\Vert {\bf{W}}-{\bf{B}}\Vert }_{F}^{2}$$

where **W** is treated as a proxy for **B**, and the fourth term is the relaxation of the equality constraint **W** = **B**. The last ingredient is to enforce the equality of **W** and **B** at convergence. A dual variable (or Lagrange multiplier) Λ is used to enforce the equality of **W** and **B** at convergence:

3 $$\mathop{{\rm{\min }}}\limits_{{\bf{W}},{\bf{B}}}\,\alpha {\Vert {\bf{W}}\Vert }_{1}+\beta \sum _{i=1}^{P}{\Vert {{\bf{w}}}^{i}\Vert }_{2}+\frac{1}{2}{\Vert {\bf{W}}-{\bf{B}}-{\boldsymbol{\Lambda }}\Vert }_{F}^{2}+\frac{1}{2}{\Vert {\bf{XB}}-{\bf{Y}}\Vert }_{F}^{2}$$

Equation (3) shows a sparse-and-group model that can obtain the optimal solution through convex optimization and ADMM. However, many studies have shown that improved performance can be obtained by using nonconvex optimization^12,13^. Thus, nonconvex optimization is used to solve our problem. Let *u* ∈ **R**, we first introduce shrinkage mappings^12^ *S*_*u*_ and **S**_*u*_ from **R**^*N*^ × **R**_+_ to **R**^*N*^:

4 $${S}_{u}{({\bf{b}},\alpha )}_{i}=\frac{{b}_{i}}{|{b}_{i}|}\,{\rm{\max }}\,\{0,|{b}_{i}|-{\alpha }^{2-u}{|{b}_{i}|}^{u-1}\}$$

5 $${{\boldsymbol{S}}}_{u}({\bf{b}},\alpha )=\frac{{\bf{b}}}{{\Vert {\bf{b}}\Vert }_{2}}\,{\rm{\max }}\,\{0,{\Vert {\bf{b}}\Vert }_{2}-{\alpha }^{2-u}{\Vert {\bf{b}}\Vert }_{2}^{u-1}\}$$

We use these two shrinkage mappings with *u* < 1. Equations (4) and (5) are an extension of soft-thresholding^20^, which appears in many sparsity-related algorithms:

6 $${S}_{1}{({\bf{b}},\alpha )}_{i}=\frac{{b}_{i}}{|{b}_{i}|}\,{\rm{\max }}\,\{0,|{b}_{i}|-\alpha \}$$

Equation (6) incorporates soft-thresholding and is the proximal mapping for the L1 norm:

7 $$\mathop{\text{arg}\,\min }\limits_{{\bf{w}}}\,\alpha {\Vert {\bf{w}}\Vert }_{1}+\frac{1}{2}{\Vert {\bf{w}}-{\bf{b}}\Vert }_{2}^{2}={{S}}_{1}\,({\bf{b}},\alpha )$$

**Figure 2:**
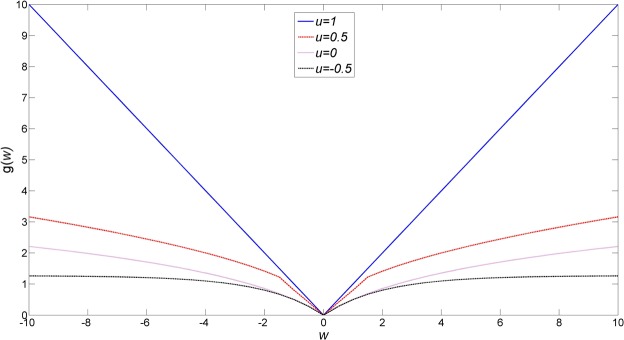
Plots for the function *g* in Eq. (9) using *α* = 1. The smaller the value of *u* is, the slower of the growth of *g*.

If there is a real-valued function *G* such that for any **w** ∈ **R**^*N*^:

8 $$\mathop{\text{arg}\,\min }\limits_{{\bf{w}}}\,\alpha G({\bf{w}})+\frac{1}{2}{\Vert {\bf{w}}-{\bf{b}}\Vert }_{2}^{2}={{S}}_{u}({\bf{b}},\alpha )$$

where $G({\bf{w}})={\sum }_{i=1}^{Q}g({w}_{i})$ and *g*(*w*) is a penalty function (see Fig. 2 for numerically computed plots; the function *g*(*w*) grows like |*w*|^*u*^/*u* + *C* for large |*w*| and some C (or $\mathrm{log}|w|+C$ for *u* = 0)), then for any **w** ∈ **R**^*N*^:

9 $$\mathop{{\rm{\arg }}\,{\rm{\min }}}\limits_{{\bf{w}}}\,\alpha g({\Vert {\bf{w}}\Vert }_{2})+\frac{1}{2}{\Vert {\bf{w}}-{\bf{b}}\Vert }_{2}^{2}={{\boldsymbol{S}}}_{u}({\bf{b}},\alpha )$$

The form of Eq. (3) that contains a nonconvex penalty can be written as:

10 $$\mathop{{\rm{\min }}}\limits_{{\bf{W}},{\bf{B}}}\,\alpha {G}_{\alpha ,{u}}({\bf{W}})+\beta \sum _{i=1}^{P}{g}_{\beta ,{v}}({\Vert {{\bf{w}}}^{i}\Vert }_{2})+\frac{1}{2}{\Vert {\bf{W}}-{\bf{B}}-{\boldsymbol{\Lambda }}\Vert }_{F}^{2}+\frac{1}{2}{\Vert {\bf{XB}}-{\bf{Y}}\Vert }_{F}^{2}$$

Finally, the original problem, which is represented by Eq. (3), is transformed into an easily solvable nonconvex optimization problem. Chartrand *et al*.^12,21^ proposed the details and a method proof. The NcADMM algorithm for sparsity with group sparsity is shown in Algorithm 1.

Using Eq. (10), we find edges between DNA methylation sites and genes, where linear loss is used because gene expression values are continuous. Next, we use Eq. (12) to find edges between genes and disease, where logistic loss is used for binary status (i.e., disease status is denoted by 1 and normal status is denoted by 0). Given a feature (i.e., gene) vector **y** as follows:

11 $$p(z=1|{\bf{y}};\theta )=\sigma ({\theta }^{T}{\bf{y}})=\frac{1}{1+\exp (\,-\,{\theta }^{T}{\bf{y}})}$$

where *θ* ∈ **R**^*Q*^ is the coefficient vector of the logistic regression model, and *σ*(⋅) is the sigmoid function, then L1-regularized logistic regression is defined as follows:

12 $$\mathop{{\rm{\min }}}\limits_{\theta }\sum _{i=1}^{N}-\,\mathrm{log}\,p({z}^{(i)}|{{\bf{y}}}^{(i)};\theta )+\lambda {\Vert \theta \Vert }_{1}$$

**Algorithm 1:**
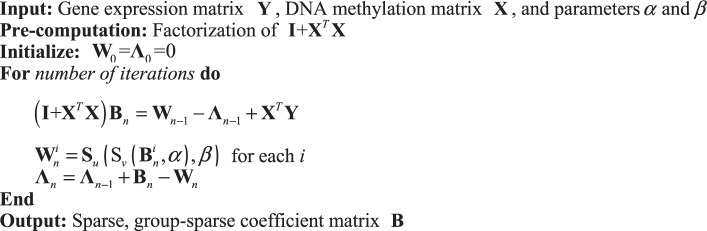
NcADMM algorithm for sparsity with group sparsity.

In practice, NAMDD is applied to one kind of disease and the disease status matrix is **Z** ∈ **R**^*N*×1^. Meanwhile, we determine the regularization parameters *α*, *β* and *λ* using cross-validation; however, with cross-validation, we often obtain many false positives (i.e., a normal DNA methylation site is identified as a disease-related methylation site). To effectively control false positives, we use NcADMM and LLR under stability selection, which shall be described in the next section.

### Calculating the edge score under stability selection

Based on the resampling technique, stability selection can effectively control false positives. We use NcADMM and LLR with a stability selection strategy to detect edges in an association network. Briefly, the stability selection procedures for NAMDD are as follows. First, we randomly select half of the number of samples *T* times and apply NcADMM and LLR to the corresponding selected datasets (i.e., NcADMM for the DNA methylation and gene expression datasets and LLR for the gene expression and disease status datasets). Second, stability selection reserves DNA methylation sites or genes whose coefficients are non-zero for *T*⋅*ϕ* times, where *ϕ* is a threshold parameter that controls the number of false positives. We summarized NAMDD under stability selection in Algorithm 2. We discuss our choice of two parameters, *T* and *ϕ*, next. Meinshausen *et al*.^18^ reported that *T* ≥ 100 is sufficient to achieve false positive control. In practice, *ϕ* is chosen to be between 0.5 and 1. The larger *ϕ* is, the better the false positive control at the cost of a decreased true positive rate. In theory^18^, under certain conditions, the relationship between the number of false positives and *ϕ* has been established. When finding edges between DNA methylation sites and the *q*-th gene trait,

13 $$E({V}_{q})\le \frac{1}{2\varphi -1}\frac{{c}_{{\alpha }^{\ast },{\beta }^{\ast }}^{2}}{P}$$

where *E*(*V*_*q*_) is the expected number of falsely detected DNA methylation sites for the *q*-th gene trait, and ${c}_{{\alpha }^{\ast },{\beta }^{\ast }}$ is the number of nonzero coefficients found by NcADMM with α^*^ and β^*^. Equation (13) shows that the upper bound of the number of false positives is inversely proportional to *ϕ*. The same situation exists when detecting associations between gene traits and disease.

We use stability selection to calculate the score of every edge in an association network. For the edge connecting the *p*-th DNA methylation site and the *q*-th gene trait edge, the score is defined as follows:

14 $$score\,({e}_{q}^{p})=\frac{\#(p,q)}{T}$$

where ${e}_{q}^{p}$ indicates the edge connecting the *p*-th DNA methylation site and *q*-th gene trait. #(*p*, *q*) indicates the number of datasets in which ${e}_{q}^{p}$ is successfully identified among all *T* datasets generated by the same parameters. The range of the score is 0 to 1. Obviously, the larger the score is, the stronger the relationship between the *p*-th DNA methylation site and the *q*-th gene trait.

Based on the edge scores, we can calculate the path scores. We assume that the path, which is composed of significant edges, is also a significant path. To effectively find significant paths, we use a path search algorithm, which shall be described in the next section.

### Using a path search algorithm to detect important path associations

There are many path associations in an association network. In order to find significant paths from DNA methylation sites to disease. Based on previous research, we use a path search algorithm to find important paths (i.e., paths with high scores) in an association network^22^. It should be noted that, significant paths tend to have large scores, and a path means a continuous pathway from a DNA methylation site to a gene and from the gene to a disease.

In an association network, the procedure for finding important paths is as follows. First, we look for all genes that are both connected to the DNA methylation sites and connected to the disease; thus, we can use these genes to find all existing paths. Second, the score of path can be obtained by summing edge scores that belong to the path. Finally, we can find *K* maximum score paths.

The path score equation is defined as follows:

15 $$score\,({\rm{Path}})={\sum }_{{{\rm{Edge}}}_{i}\in {\rm{Path}}}score\,({{\rm{Edge}}}_{i})$$

**Algorithm 2:**
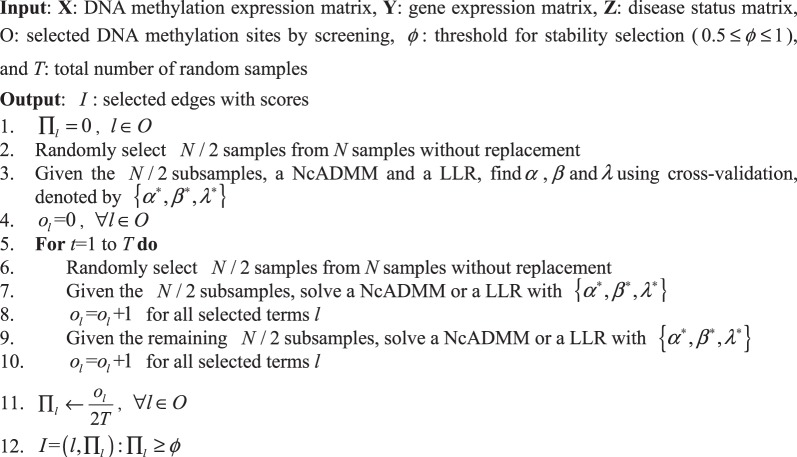
NAMDD under stability selection.

### A screening strategy based on NAMDD

In the process of finding significant path associations, stability selection requires multiple runs of the NcADMM algorithm. This is particularly problematic when finding edges (i.e., associations) between DNA methylation sites and genes in large-scale cancer datasets (e.g., *P* = 450000 DNA methylation values from the Illumina Infinium HumanMethylation450k platform); thus, in this section, we propose a screening method to improve the efficiency of the NcADMM algorithm:

16 $$\mathop{{\rm{\min }}}\limits_{{b}_{j}}\frac{1}{2}{\Vert {\bf{Y}}-{{\bf{x}}}_{j}{{\bf{b}}}_{j}\Vert }_{F}^{2}$$

where **Y** ∈ **R**^*N*×*Q*^ is the entire gene expression data matrix, **x**_*j*_ ∈ **R**^*N*^ is the expression values for the *j*-th DNA methylation site among *N* samples, **b**_*j*_ ∈ **R**^*Q*^ is the coefficient vector for the *j*-th DNA methylation site corresponding to its effect size on *Q* genes, and each entry corresponds to the effect of the *j*-th DNA methylation site on a gene.

The idea of the screening method is to retain DNA methylation sites that have strong associations with gene traits. We consider one DNA methylation site at a time. After calculating the coefficient vector for each DNA methylation site, we put all the coefficient vectors together to form a matrix **L** ∈ **R**^*P*×*Q*^ whose rows correspond to DNA methylation sites and columns correspond to genes. After the screening process, we select the top *d* DNA methylation sites for each gene based on the absolute value of the coefficients and then apply NAMDD to the filtered dataset. This strategy is similar to the single variable screening step followed by joint analysis in linear regression. For large-scale cancer datasets (e.g., *P* = 450000 DNA methylation values from the Illumina Infinium HumanMethylation450 k platform), we recommend using this screening method to reduce the number of DNA methylation sites.

### Synthetic datasets and ovarian cancer data

We generated eight kinds of simulation datasets with different sample sizes, different numbers of methylation-gene true associations and the same number of features (i.e., 1000 DNA methylation sites, 100 genes, and 1 disease status). Here, 150 methylation site-10 gene true associations, which are present in *N* ∈ {200, 500, 800, 1100} samples, were used to introduce the data generation process. In the four other simulation datasets, 300 methylation sites linked to 30 genes were used to show true associations. First, we generated 150 causal DNA methylation sites that are actually related to disease. The disease status is a balanced number case-control status (i.e., equal numbers of 0 s and 1 s, 0 is used to indicate a normal status, and 1 is used to indicate a disease status). Second, a three-layer neural network was used to generate 10 gene expression levels, where adjacent layers are fully connected. In the three-layer neural network, the input layer with 150 nodes represents DNA methylation sites, the middle layer with 10 nodes represents gene traits, and the output layer with 1 node represents the disease status. Third, the three-layer neural network was trained until more than 95% of disease status nodes were correctly predicted using a back propagation (BP) algorithm implemented using Tensor Flow^23^. After training, the values in the middle layer nodes were used as gene expression values. Finally, for each sample, we added 850 DNA methylation site values drawn from *N*(0,1) and 90 gene expression values drawn from a Gaussian distribution with the same variance as the 10 gene expression levels to include DNA methylation sites and gene traits not associated with the disease pathogenesis mechanism. We also added noise data from *N*(0,1).

Ovarian cancer data from TCGA^24^. DNA methylation profile of TCGA ovarian cancer data was measured experimentally using the Illumina Infinium HumanMethylation27 platform by the Johns Hopkins University and University of Southern California TCGA genome characterization center. The gene expression profile was measured experimentally using the Affymetrix HT-HGU133A platform by the Broad Institute of MIT and Harvard University cancer genomic characterization center. The disease status data was derived at the Broad Institute of MIT and Harvard University cancer genomic characterization center.

We compared our method NAMDD with two widely used methylation site search methods NsRRR-Logistic and PCLOGIT. We evaluated their performance in detecting disease-related DNA methylation sites.

For NsRRR-Logistic, we first used NsRRR to identify DNA methylation sites associated with gene traits. These DNA methylation sites were identified as being associated with disease, and L1-regularized logistic regression was used to evaluate the significance of the disease-related DNA methylation sites. We then determined the NsRRR and L1-regularized logistic regression regularization parameters using a 10-fold cross-validation strategy. Finally, we followed the NsRRR strategy to set the prior knowledge parameters for DNA methylation sites and genes in NsRRR. The prior knowledge setting strategy for NsRRR is as follows. In a DNA methylation network, NsRRR uses signal-carriers to represent disease-related methylation sites and non-signal-carriers to represent normal methylation sites; thus, *p*_*C*_ = 0.4 represents the probability of a connection between signal-carriers (i.e., disease-related methylation sites) in the same sub-network, *p*_*CC*_ = 0.13 represents the probability of a connection between single-carriers or non-signal-carriers in different sub-networks, and *p*_*SN*_ = 0.04 is the probability of a connection between a signal-carrier and a non-signal-carrier. The same prior knowledge parameters are present in the corresponding gene network. We applied NsRRR-Logistic to synthetic datasets using the above parameters.

PCLOGIT is a penalized conditional (unconditional) logistic regression method that uses a network-based penalty for matched (unmatched) case-control data. For PCLOGIT, we used the R package ‘PCLOGIT’ with default settings. The function ‘sel.pclogit’ from the R package returns the selection probabilities of methylation sites, which were computed based on a resampling strategy. Thus, we could use selection probabilities to calculate the true positive rate (TPR), false positive rate (FPR) and area under the curve (AUC).

**Figure 3:**
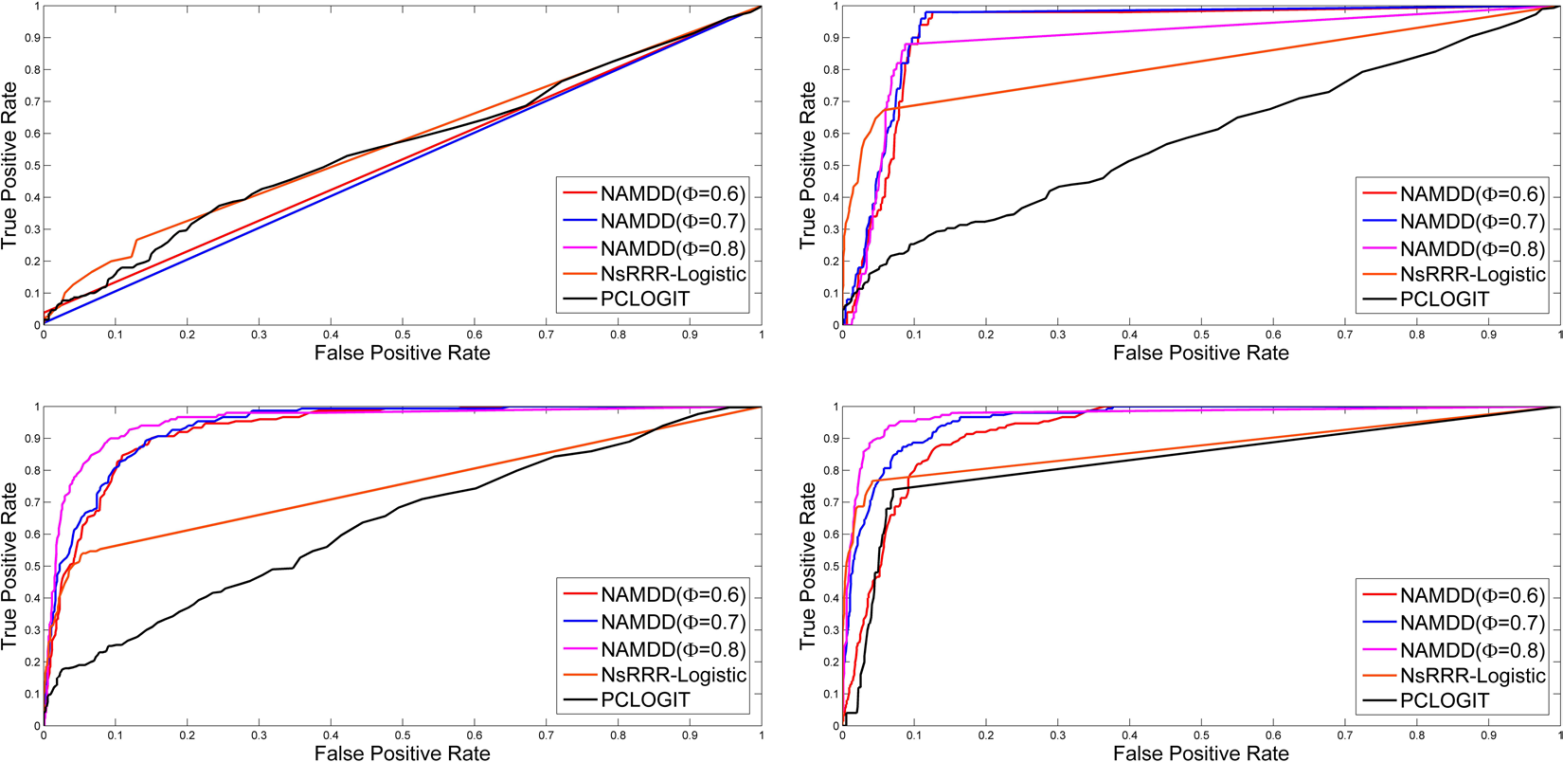
ROCs of NAMDD, NsRRR-Logisitc and PCLOGIT in 150 methylation sites-10 genes true associations simulation datasets. *N* = 200 (top left), *N* = 500 (top right), *N* = 800 (bottom left), and *N* = 1100 (bottom right). For NAMDD, we show the results with three settings for *ϕ* from 0.6 to 0.8.

**Figure 4:**
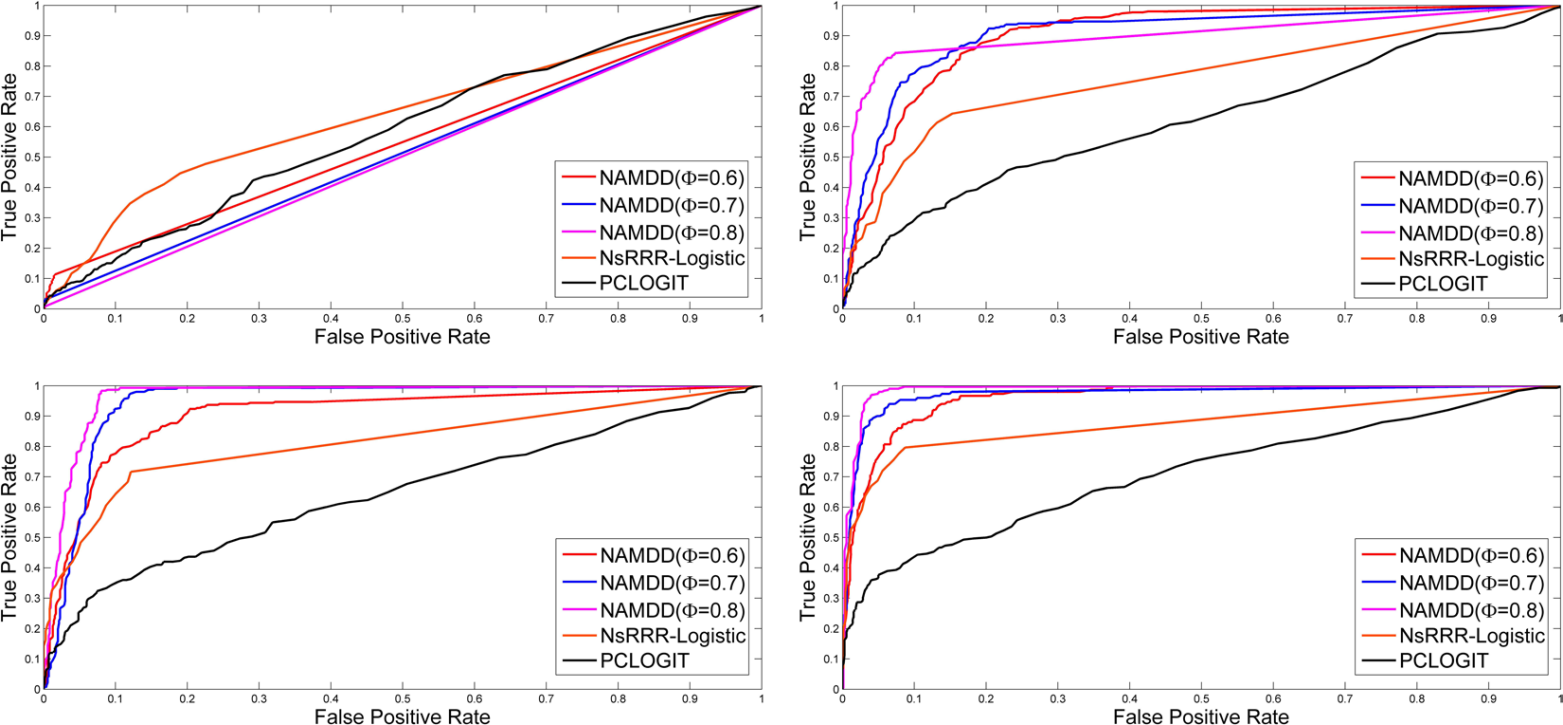
ROCs of NAMDD, NsRRR-Logisitc and PCLOGIT in 300 methylation sites-30 genes true associations simulation datasets. *N* = 200 (top left), *N* = 500 (top right), *N* = 800 (bottom left), and *N* = 1100 (bottom right). For NAMDD, we show the results with three settings for *ϕ* from 0.6 to 0.8.

For NAMDD, we changed *ϕ* from 0.6 to 0.8, set *T* = 100, u = 0.5 and v = 0.5 and selected up to *K* = 2000 paths. In Figs 3 and 4, the ROC curves show the TPRs and FPRs of the results produced by NAMDD with three different parameter settings *ϕ* = {0.6,0.7,0.8}, NsRRR-Logistic, and PCLOGIT. Each panel shows the results for different sample sizes and different numbers of methylation-gene true associations from *N* = 200 to *N* = 1100. The corresponding AUC values and ACC (accuracy) values from Figs 3 and 4 are shown in Supplementary Tables S1 and S2, respectively.

All experiments were performed on the same computer with an Intel Xeon E5-2650 CPU and 128 G RAM; we received a MATLAB license from the school software service center (http://software.tongji.edu.cn/).

### True-signatures test

In true-signatures test, we first use a conventional linear regression method FaST-LMM-EWASher^25^ (Factored Spectrally Transformed Linear Mixed Model for Epigenome-Wide Association studies) to predefine OC-associated DNA methylation sites which are defined as true signatures. We then compare the performance of three methods in picking up these true signatures. A good disease-related methylation analysis method should report as many as true signatures as possible, and at the same time report as less methylation sites as possible.

### Sample exchange test

In the designed sample-exchange test, disease-related methylation analysis is firstly conducted on the original dataset with correct sample class label and generated a set of “actual” result; then disease-related methylation analysis is applied to a “mock” dataset with the samples exchanged between two class conditions to generate a set of “mock” result. Compared with the “actual” result that is expected to carry biological meaning, the “mock” result in generated with wrong sample labels and thus represents a background associated with no biological meanings. A good disease-related methylation analysis method should report as many as disease-related methylation sites (DMSs) as possible in the “actual” result, and at the same time report as less DMSs as possible in the “mock” result given a specific stability selection score. When two methods report the same number of DMSs on the “mock” dataset, the one that reports more DMSs on the “actual” dataset achieved a better performance.

### Estimating significance of paths

We assessed the statistical significance of an observed path (OP) from the top 22 paths by comparing its path score (PS) with the set of scores PS_NULL_ computed with randomly assigned data^26,27^. First, we randomly assign original methylation samples, randomly arrange original gene samples, randomly assign original disease status labels to samples, and re-compute the PS of an OP. Second, we repeat the previous step with 1000 permutations and create a histogram of the corresponding path scores PS_NULL_. Third, we estimate the P-value for an OP from PS_NULL_ by calculating the fraction of the 1000 random permutations in which the OP gave a smaller PS than that observed in the original data.

In our study, the null hypothesis is that the score of the path is random with regard to the sample categorization. The alternative hypothesis is that the score of the path is associated with specific diagnostic criteria used to assign labels to samples. Suppose the P-value of the path association is 0.008, which means that there are eight permutation test results smaller than the original path score under the null hypothesis.

## Results

In this section, we first compared the performance of NAMDD with those of two well-known methods (i.e., NsRRR and PCLOGIT) using synthetic data sets; the results show that NAMDD outperforms the other methods. The boxplots show that the stability of NAMDD is better than the other methods. NAMDD achieved a better performance in real ovarian cancer data sample-exchange test. We then applied NAMDD to the ovarian cancer data from TCGA and identified 389 significant path associations, among which we analyzed the statistical significance of DNA methylation sites and genes from top 22 paths. The statistical significance of the top 22 path associations were estimated using a permutation test.

### Performance comparison on synthetic data

**Figure 5:**
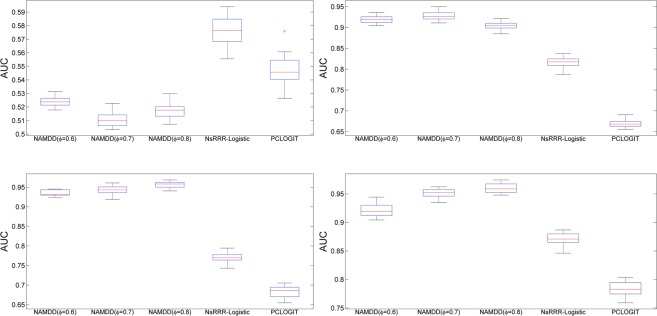
The boxplots of the AUCs for NAMDD, NsRRR-Logistic and PCLOGIT with different sample sizes. *N* = 200 (top left), *N* = 500 (top right), *N* = 800 (bottom left), and *N* = 1100 (bottom right). Here, 150 methylation sites linked to 10 genes were used to show true associations in four simulation datasets.

**Figure 6:**
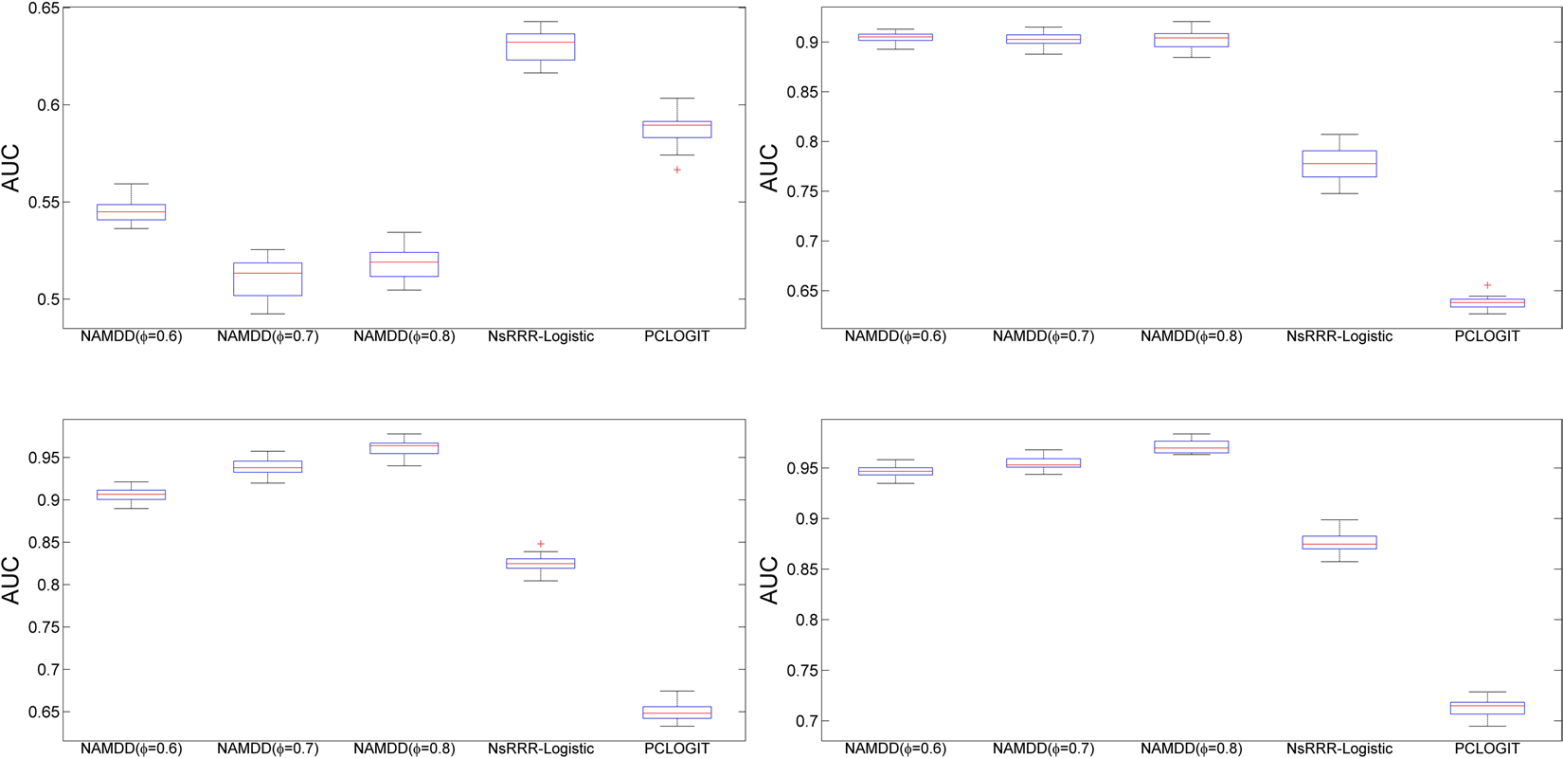
The boxplots of the AUCs for NAMDD, NsRRR-Logistic and PCLOGIT with different sample sizes. *N* = 200 (top left), *N* = 500 (top right), *N* = 800 (bottom left), and *N* = 1100 (bottom right). Here, 300 methylation sites linked to 30 genes were used to show true associations in four simulation datasets.

Compared to NsRRR-Logistic and PCLOGIT, NAMDD showed significantly better performance (larger area under the curve) for *N* > 200 regardless of the setting for *ϕ*. The results suggest that when the disease pathogenesis mechanism is complex such that DNA methylation sites affect a disease via multiple layers, two-way association (i.e., DNA methylation sites and disease or DNA methylation sites and gene traits) analysis can be ineffective in capturing causal DNA methylation sites. For each kind of dataset (e.g., *N* = 200, 150 methylation site-10 gene true associations), we generated these kind of datasets 20 times, calculated AUC values for all methods using the datasets and generated their boxplots. The boxplots are shown in Figs 5 and 6. The boxplots show that the stability of NAMDD is better than those of NsRRR and PCLOGIT.

### Performance comparison on ovarian cancer data

Ovarian malignancy is one of the common malignant tumors in the female genital organs. Because ovarian cancer (OC) has no symptoms during the early stages of the disease, it is difficult to identify whether the tumor is benign or malignant. Many patients are diagnosed after ovarian cancer has metastasized. Recent studies have shown that aberrant DNA methylation plays an important role in the malignant cell process^28,29^. Identifying path associations (i.e., DNA methylation sites to disease through gene expression traits) in ovarian cancer could yield insights into the complex epigenetic mechanisms affecting cancer.

We applied NAMDD, NsRRR-Logisitc and PCLOGIT to the TCGA ovarian cancer data, containing measurement profiles of both DNA methylation and gene expression for 592 samples. This dataset includes 24862 DNA methylation sites and the expression values for 12043 DNA probes from the same samples including known and predicted genes. For ovarian cancer disease status, we used binary classification labels (i.e., 0 for normal and 1 for disease).

**Figure 7:**
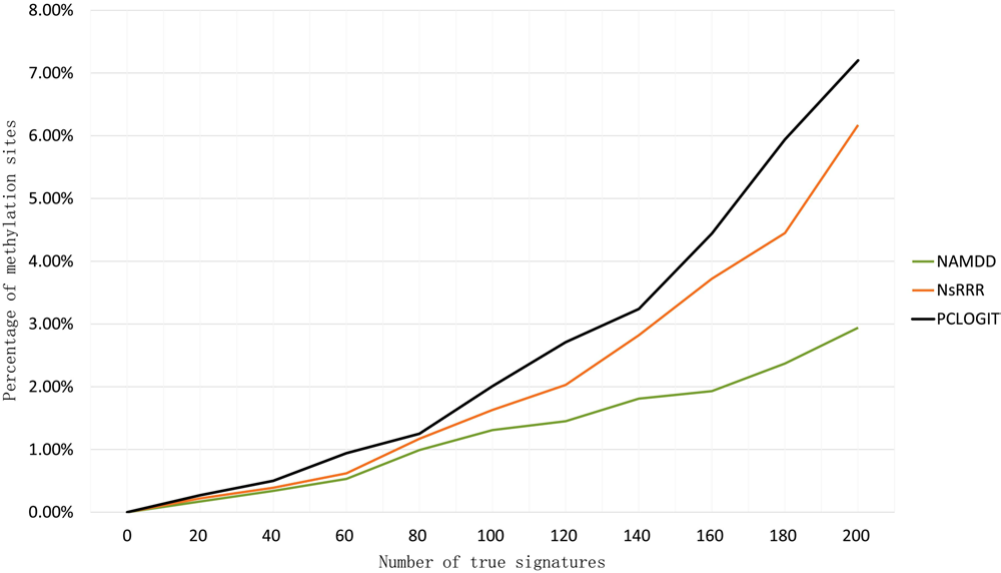
Comparison of three methods on ovarian cancer data experiment with true-signatures test. NAMDD outperforms the competing methods.

A major limitation for testing disease-related methylation site analysis methods with real dataset is the lack of experimentally validated true disease-related methylation sites. Without ground truth, it is difficult to effectively compare the performance of different approaches. For this reason, we first used FaST-LMM-EWASher to predefine OC-associated DNA methylation sites (true signatures). Because we expect some, but not too many methylation sites to be related with OC. We selected the top 200 sites from the results of the FaST-LMM-EWASher according to FaST-LMM-EWASher usage and threshold criteria (P-value < 2.02e-20, Q-value < 6.46e-19). We then compared the performance of three methods in picking up these true signatures. In the Fig. 7, x-axis represents the number of true signatures, and y-axis represents the percentage of methylation sites containing true signatures. The detail information of Fig. 7 is shown in Supplementary Table S3.

We designed a sample-exchange test to compare the performance of different approaches by taking advantage of a true null data generated by sample exchange. Such kind of evaluations are widely used in bioinformatics studies to compare performance of different methods when the ground truth is not known^30–33^.

As is shown in Supplementary Figure 1, NAMDD outperforms the other methods on real disease-methylation dataset in sample exchange test. In the Supplementary Figure 1, x-axis represents the percentage of DMSs found in the “mock” dataset, and y-axis represents the percentage of DMSs found in the “actual” dataset. For NAMDD, when 0.5% of sites are reported as DMSs on the “mock” dataset, around 2.5% of DMSs are reported on the corresponding “actual” dataset. The detail information of Supplementary Figure 1 is shown in Supplementary Table S4.

### Application to ovarian cancer data

We ran Algorithm 2 with a 10-fold cross-validation,*ϕ* = 0.6, and *T* = 100. We chose *ϕ* = 0.6 to ensure that all potentially interesting paths were included in the results. We found 389 paths in the OC data. The paths involve a DNA methylation site, a gene, and the disease. To the best of our knowledge, OC-related path associations have not been reported in the previous literature. The maximum path score is 2. We focused on analyzing the top 22 paths because of these paths are the highest-scoring (Table 1. column 6) paths containing three known ovarian cancer oncogenes *CCNE1*, *AURKA* and *RAB25*^34–36^. The 22 path associations and corresponding path P-values (Table 1. column 7) are shown in Table 1.

**Table 1:** Top 22 path associations found by NAMDD in the OC data related to *CCNE1*, *AURKA* and *RAB25* genes.

DNA methylation site	DNA methylation site position	Annotation	Genes nearby DNA methylation site within 1 Mbp	Gene	Gene start position	Path score	Path P -value
cg20822628	chr20:61041592	gene body	*GATA5*	*CCNE1*	chr19:30302805	1.97	0.011
cg00499822	chr10:44881551	gene body	*CXCL12*	*CCNE1*	chr19:30302805	1.96	0.01
cg23588759	chr6:88039288	gene body	*GJB7*; *SMIM8*	*CCNE1*	chr19:30302805	1.95	0.009
cg03504078	chr5:140480218	Intergenic	*AC005754*	*CCNE1*	chr19:30302805	1.95	0.012
cg08861115	chr2:113735377	gene body	*IL36G*	*CCNE1*	chr19:30302805	1.92	0.008
cg21906716	chr1: 3579978	gene body	*TP73*	*CCNE1*	chr19:30302805	1.91	0.017
cg19290962	chr20:2517613	promoter	*TMC2*	*CCNE1*	chr19:30302805	1.90	0.009
cg16935609	chr5:6632086	promoter	*NSUN2*; *SRD5A1*	*CCNE1*	chr19:30302805	1.82	0.033
cg09111917	chr3:44915918	promoter	*TGM4*	*CCNE1*	chr19:30302805	1.81	0.015
cg01309153	chr9:136224666	Intergenic	*SURF1*	*CCNE1*	chr19:30302805	1.80	0.021
cg16391792	chr15:58723657	gene body	*ALDH1A2*	*AURKA*	chr20:54944445	1.96	0.021
cg05697231	chr15:74286614	promoter	*PML*	*AURKA*	chr20:54944445	1.95	0.016
cg19515446	chr6:26108335	gene body	*HIST1H1T*	*AURKA*	chr20:54944445	1.92	0.02
cg19035993	chr22:32108701	gene body	*PRR14L*	*AURKA*	chr20:54944445	1.92	0.03
cg04711324	chr18:40695633	gene body	*RIT2*	*AURKA*	chr20:54944445	1.90	0.021
cg17675150	chr18:56529784	promoter	*ZNF532*	*AURKA*	chr20:54944445	1.86	0.15
cg19320612	chr2:167168190	gene body	*SCN9A*	*AURKA*	chr20:54944445	1.83	0.018
cg22313025	chr6:105307096	gene body	*HACE1*	*RAB25*	chr1:156030951	1.96	0.02
cg23303782	chr10:120967744	gene body	*GRK5*	*RAB25*	chr1:156030951	1.92	0.018
cg17749520	chr17:42466567	gene body	*ITGA2B*	*RAB25*	chr1:156030951	1.91	0.01
cg08377000	chr4:90033921	promoter	*FAM13A*;*TIGD2*	*RAB25*	chr1:156030951	1.87	0.011
cg03381111	chr15:25296571	gene body	*SNHG14*	*RAB25*	chr1:156030951	1.84	0.025

Path P-values were obtained from permutation test.

#### Significant analysis of DNA methylation sites and gene in independent data

Having identified 22 significant methylation sites, we attempted to replicate these methylation significances in independent data (GSE15373) from ovarian cancer cases and controls. The methylation profile was measured experimentally using the genome tiling array by the Indiana University medical sciences. To verify whether the methylation sites are specifically functions in the disease, we used Student’s t test to calculate P-values of DNA methylation sites from 22 paths. The results are shown in Table 2. As is shown in Table 2, Student’s t-test P-values for all 22 DNA methylation sites are less than 0.01, which means that we can reject the null hypothesis and consider DNA methylation sites of 22 paths are differentially expressed between normal and cancer.

**Table 2:** The Student's t-test P-values and T-scores of DNA methylation sites from 22paths.

DNA methylation site	P-value	T-score (case-control)
cg20822628	7.88e-28	−17.014
cg00499822	6.95e-10	−5.332
cg23588759	2.69e-21	−10.521
cg03504078	7.93e-31	−3.614
cg08861115	3.58e-35	−19.391
cg21906716	9.50e-28	−16.988
cg19290962	4.15e-15	−13.211
cg16935609	1.82e-36	−11.040
cg09111917	2.88e-44	−9.877
cg01309153	6.95e-05	−13.466
cg16391792	6.18e-24	−10.893
cg05697231	7.38e-03	−3.056
cg19515446	4.74e-14	−12.780
cg19035993	7.15e-15	−4.639
cg04711324	3.12e-15	−13.973
cg17675150	8.57e-20	−20.089
cg19320612	5.91e-38	−14.561
cg22313025	8.49e-05	−5.722
cg23303782	4.83e-12	−4.771
cg17749520	6.46e-08	−3.592
cg08377000	6.79e-11	−15.435
cg03381111	1.57e-17	−6.131

We used Pearson correlation coefficient (PCC) to calculate correlation coefficients of DNA methylation sites and genes in disease samples. The results are shown in Supplementary Table S3. The PCCs and corresponding P-values provided in Supplementary Table S3 indicate that these DNA methylation site levels are significantly negatively correlated with gene express level. Based on the above mentioned information, we are confident that the changes of DNA methylation level in the sites involved in the 22 paths are negatively correlated with gene express level when comparing the case of disease and normal. The PCCs and corresponding P-values from 389 paths provided in Supplementary Table S7.

Based on the representative work of transcription factor research^37–39^, we collected 2574 transcription factor genes and calculated the proportion of transcription factor genes near these DNA methylation sites. We found that 6 of 22 DNA methylation sites are located nearby these genes. In order to check if these genes are in fact over represented, we used GSEA software (http://software.broadinstitute.org/gsea/index.jsp) for gene set enrichment analysis (GSEA)^40^. The |NES| > 1, NOM p-val < 0.001, and FDR q-val < 0.05. The GSEA results indicate that these genes are over represented. The detailed location information of 6 DNA methylation sites and transcription factor genes GSEA results are provided in Supplementary Table S6.

Having identified 3 significant genes, we attempted to replicate these gene expression differences in independent data (GSE14407) from ovarian cancer cases and controls. The gene expression profile was measured experimentally using the Affymetrix U133 Plus 2.0 platform by the Clark Atlanta University cancer research and therapeutic development center. We used Student’s t test and Wilcoxon rank sum test to estimate the significance of differential expression. The results of Student’s t test and Wilcoxon rank sum test are shown in Table 3. In Student’s t test, the P-values of three genes are less than 0.01. Meanwhile, in Wilcoxon rank sum test, the P-values of three genes are less than 0.01, and H-values are equal to 1. The results of Student’s t test and Wilcoxon rank sum test show that genes from 22 paths are significantly differentially expressed between normal and cancer.

**Table 3:** The Student’s t test and Wilcoxon rank sum test for genes from 22 paths.

Method value	*CCNE1*	*AURKA*	*RAB25*
Student’s t test P-value	2.17e-06	5.42e-11	7.15e-04
Student’s t test T-score (case-control)	21.9653	26.4098	7.4899
Wilcoxon rank sum test P-value	2.64e-08	1.98e-07	2.54e-05
Wilcoxon rank sum test H-value	1	1	1

We first perform genome-wide differential expression analysis using the conventional approaches, and then see how many of these differentially expressed genes are picked up by the 3 Methods. We used a classic epigenome-wide differential expression analysis method edgeR^41^ (Empirical analysis of Digital Gene Expression in R) to find differentially expressed genes. We then compared the performance of three methods (NAMDD/NsRRR/PCLOGIT) in picking up these differentially expressed genes. Among the 100 differentially expressed genes found by edgeR, the results of NAMDD included 52 genes, NsRRR found 34 genes, and PCLOGIT found 15 genes.

## Discussion

In this section, we first tried to investigate and explain the biological mechanisms of path associations based on bioinformatics databases and our extensive literature survey. Further biological studies are required to confirm our proposed hypotheses. In other paths not discussed in the article, three genes were involved including *RFC4*, *TPX2*, and *ASNS*. We found no reported associations between these genes and OC. However, these genes are related to breast cancer^42–46^. In future work, it would be interesting to investigate the relationships between these genes and OC. We then discussed the experimental results and work’s extensions.

### Path associations containing CCNE1

We identified 10 path associations that involve cyclin e1 (*CCNE1*). *CCNE1* is a protein coding gene according to GeneCards (www.genecards.org)^47–49^ and the human protein-protein interaction database^50–54^. *CCNE1* encodes cyclin e1 protein. This cyclin forms a complex with and functions as a regulatory subunit of *CDK2*, whose activity is required for the cell cycle G1/S transition. Thus, *CCNE1* promotes progression of the cell from the G1 to the S phase of the cell cycle^55^ and is a proliferation marker^56^. Many studies have reported that this gene is associated with cancers^57–59^, by contributing to tumor genesis. Next, we tried to investigate and explain the cancer-related regulation mechanisms underlying path associations between the DNA methylation sites and *CCNE1* in 2 of 10 path associations.

**Figure 8:**
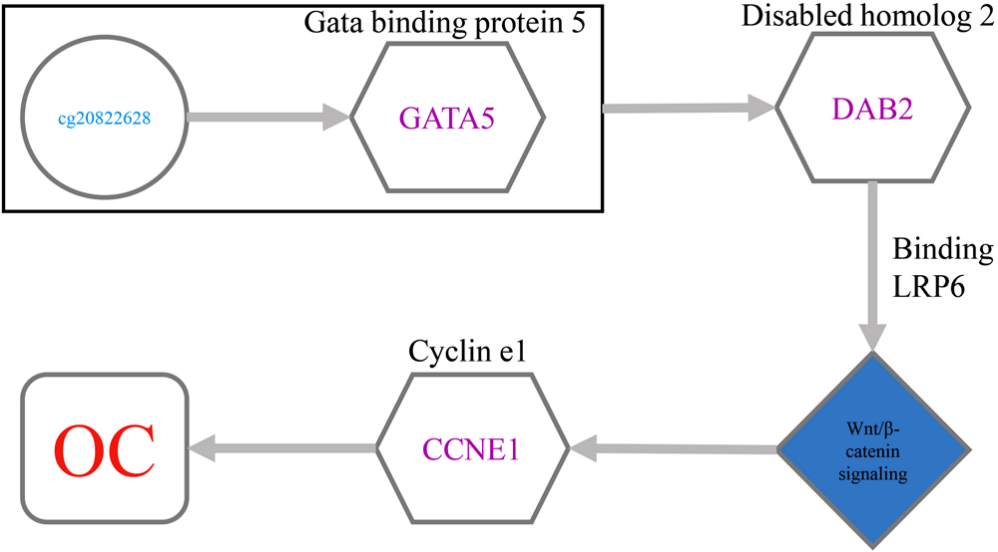
Hypothetical pathway for the path association involving cg20822628 (close to GATA5), CCNE1 and OC.

In the path association cg20822628 → *CCNE1* → OC, cg20822628 (chr20: 62466536–62466537) is located 9435 bp downstream of the transcriptional start site (TSS) of gata binding protein 5 (GATA5). Research shows that the transcriptional silencing of *GATA5* causes silencing of *DAB2* (disabled homolog 2)^60^. The PCC between *GATA5* and *DAB2* and corresponding P-values are −0.5604 and 0.0118, respectively. *DAB2* is a potential tumor suppressor^61,62^. *DAB2* and *CCNE1* are a tumor suppressor gene and oncogene pair normally involved in strong stabilizing molecular interaction negative feedback loops, and it is these interactions that are sufficiently perturbed during cancer development^63^. *DAB2* encodes a mitogen-responsive phosphoprotein that inhibits Wnt/β-catenin signaling by binding LRP6 (lipoprotein receptor related protein 6) and promoting its internalization through clathrin^64^. Activated Wnt/β-catenin signaling promotes the progression of tumor cell cycle and cell proliferation; concomitantly, the mRNA levels of *CCNE1* are higher than normal^65^. Figure 8 illustrates our hypothesis for the path association. Combining the information mentioned above, we hypothesize the ovarian cancer-related regulation mechanism as follows. Initially, the DNA methylation site cg20822628 causes the transcriptional inactivation of GATA5, which drives down-regulation or silencing of the tumor suppressor gene *DAB2*. Furthermore, low expression of the tumor suppressor gene *DAB2* enhances Wnt/β-catenin signaling activity. Additionally, active Wnt/β-catenin signaling results in high *CCNE1* expression. Finally, the high level of *CCNE1* promotes progression of the cell from the G1 to the S phase of the cell cycle, which increases the risk of OC.

In the above paragraph, we investigated and explained how the DNA methylation site promotes oncogene *CCNE1* overexpression and increases the risk of OC. In this section, we try to explain ovarian cancer cell growth and survival through a significant path. In the path association cg00499822 → *CCNE1* → OC, cg00499822 (chr10: 44386103–44386104) is located 1010 bp upstream of *CXCL12* (c-x-c motif chemokine ligand 12). The protein encoded by this gene is the ligand for the G-protein coupled receptor, chemokine (c-x-c motif) receptor 4, and plays a role in many diverse cellular functions, including inflammation response, immune surveillance and tumor growth and metastasis^66,67^. A large number of studies have shown that *CXCL12* and *CXCR4* (c-x-c motif chemokine receptor 4, *CXCL12*’s specific receptor) are involved in tumorigenesis, proliferation and metastasis^68,69^. *CXCL12*/*CXCR4* can regulate signaling pathways by altering the chemical structure of G protein. The PCC between *CXCL12* and *CXCR4* and corresponding P-values are 0.7279 and 0.0055, respectively. *CXCL12* and *CXCR4* activate the mitogen-activated protein kinase (*MAPK*), stress-activated protein kinase/Jun N-terminal kinase (SAPK/JNK), extracellular-regulated kinase 1/2 (ERK1/2) and phosphatidyl inositol 3-kinase (PI3K) pathways^70–72^. The PI3K pathway can lead to the amplification of *CCNE1* in cancer^72^. Combining the above mentioned information, we hypothesize the cancer-related DNA methylation regulation mechanism as follows. First, the DNA methylation site cg00499822 activates *CXCL12*, and the *CXCL12* and *CXCL4* interaction activates the relevant signaling pathways (i.e., MAPK, ERK1/2 and PI3Ksignaling pathways). Next, the PI3K pathway leads to *CCNE1* overexpression which leads to ovarian cancer cell growth and survival. To the best of our knowledge, our finding suggests that this pathway increases the risk of ovarian cancer. This is a newly discovered pathway associated with ovarian cancer.

### Path associations containing AURKA

We identified 7 path associations that involve *AURKA* (aurora kinase a). According to the literatures^73–76^, the protein encoded by this gene is an important serine/threonine kinase responsible for regulating cell mitosis^53^. *AURKA* causes genome instability in a variety of tumor cells. *AURKA* plays an significant role in ovarian cancer cell proliferation^77^. Next, we tried to investigate the biological mechanisms underlying associations between the DNA methylation sites and ovarian cancer with *AURKA*.

In the path association cg16391792 → *AURKA* → OC, cg16391792 (chr15: 58431458–58431459) is located 42456 bp downstream of the TSS of aldehyde dehydrogenase 1 family member a2 (*ALDH1A2*). It has been reported that *ALDH1A2* is a candidate tumor suppressor gene in prostate cancer^78^. *ALDH1A2* encodes an enzyme responsible for synthesis of retinoic acid (RA), and *RA* is an important active derivative of vitamin A which plays an important role in the control of cell differentiation and proliferation. *RA* can prevent epithelial and mesenchymal tumor formation and inhibit the growth of different tumors^79^. The PCC between *ALDH1A2* and *RA* and corresponding P-values are 0.5321 and 0.0193, respectively. *RA* induces a moderate decline in *IGF* (insulin-like growth factors) concentrations^80^ and high *IGF* levels drive overexpression of *AURKA*^81^. Combining the above mentioned information, we hypothesize the biological regulation mechanism as follows. First, the cg16391792 site causes defects in *ALDH1A2* and leads the low expression of *RA*. Second, the low expression of *RA* results in the high levels of *IGF*. Finally, the high levels of *IGF* lead to overexpression of *AURKA* and promote ovarian cancer cell proliferation.

### Path associations containing RAB25

We identified 5 path associations that involve *RAB25* (rab25, member ras oncogene family). The protein encoded by this gene is a member of the *RAS* (rat sarcoma) family of small GTPases^82^. *RAB25* is related to the proliferation, survival, migration and invasion of ovarian cancer cell according to the literature and human gene database^83,84^. *RAB25* prevents apoptosis and anoikis, including that induced by chemotherapy, and increases aggressiveness of ovarian cancer cells *in vivo*^85^.

In the path association cg22313025 → *RAB25* → OC, cg22313025 (chr6: 104859221–104859222) is located 699 bp downstream of the TSS of hect domain and ankyrin repeat containing e3 ubiquitin protein ligase 1 (HACE1). *HACE1* specifically recognizes and ubiquitinizes autophagic receptor *OPTN* (Optineurin). The *HACE1-OPTN* axis increases autophagic flux of intracellular autophagy and inhibits proliferation of tumor cell^83^. The PCC between *HACE1* and *OPTN* and corresponding P-values are 0.6059 and 0.0121, respectively. The *OPTN* and *RAB25* have an opposite effect in the autophagy mechanism^52,86^. Based on the above mentioned information, we hypothesize the biological regulation mechanism as follows. First, the cg22313025 site silences expression of the *HACE1*. Second, the silenced *HACE1* disturbs the interaction mechanisms of the *HACE1* and *OPTN*. Third, the perturbed *OPTN* level leads to high level of *RAB25*. Finally, the high level of *RAB25* prevents apoptosis, promotes cancer cell proliferation, and increases the risk of ovarian cancer.

In this article, we proposed a novel method NAMDD to discover path associations among DNA methylation sites, gene traits, and disease. We employed an ADMM nonconvex optimization algorithm to find associations considering all DNA methylation sites and all genes simultaneously, employed LLR to find associations considering all genes and disease simultaneously and used a stability selection strategy and a path search algorithm for false positive control and important path selection, respectively. At the same time we proposed a screening strategy for large-scale cancer datasets. In simulation studies we have demonstrated that our proposed approach outperforms the existing main-stream methods NsRRR-Logistic and PCLOGIT for *N* > 200 regardless of the setting for *ϕ*. The boxplots show that the stability of NAMDD is better than those of the other methods. Meanwhile, in the analysis of ovarian cancer data from TCGA, NAMDD found 389 significant path associations, among which, we investigated and explained the disease pathogenesis mechanisms, including the *CCNE1*, *AURKA*, and *RAB25* genes. True-signatures test and sample-exchange test were used to estimate the statistical significance of these paths. We also found several new cancer-related genes that should be verified through biological studies in the future.

Here, we primarily focus on discussing the results from the synthetic datasets when *N* = 200. For smaller samples (*N* = 200), the simulations suggest that NAMDD may not perform as well as the other methods. The reason is that too few training samples may lead to over-fitting of our model. Meanwhile, the largest AUC value (NsRRR-Logistic, 300 methylation sites linked to 30 genes were used to show true associations) for the sample size of 200 is 0.6368, which is shown in Supplementary Table S2. This result indicates that small samples may limit the effectiveness of all methods.

The Illumina Infinium HumanMethylation450 k platform reports DNA methylation values for approximately 450 k sites in the human genome. The data from the HumanMethylation27 k and HumanMethylation450 k platforms have the same form, meaning that NAMDD can also be applied to data from the HumanMethylation450 k platform. Unfortunately, the number of OC samples from the HumanMethylation450 k platform of TCGA is too small (*N* = 10) to be used to train a model. In the future, when the number of samples from the HumanMethylation450 k platform is sufficient, we can apply NAMDD to data from the HumanMethylation450 k platform. Meanwhile, to ensure the scalability of NAMDD to 450 k datasets or larger-scale datasets, we proposed a screening method that was introduced in the “A screening strategy based on NAMDD” section, to improve the efficiency of the algorithm. For large-scale datasets, we recommend using this screening method to reduce the number of DNA methylation sites.

In sections *Path associations containing CCNE1*, *Path associations containing AURKA* and *Path associations containing RAB25*, based on database search and literature survey, we speculated some hypothetical pathways exist among DNA methylation sites, genes and disease. It will be better to use our proposed method to identify all the intermediate nodes in the hypothetical pathways. However, we cannot identify all the intermediate nodes because of the lack of the parallel data of complete multiple omics. In the future, when the pathway-related data is complete, we will apply NAMDD to the data and verify the intermediate nodes in the hypothetical pathways.

Underlying true mechanisms are much more complicated than what we assumed, gene-gene association has received extensive attention in disease research. In this paper, the method we proposed is devoted to discovering paths from a DNA methylation site to a gene and from the gene to a disease. In the future, we will study and try to propose a method for studying gene-gene association and optimal number of associations in methylation-disease research.

Finally, it should be noted that real-world biological mechanisms involve many factors such as microRNAs, lncRNAs, protein-protein interactions and environmental factors. In the future, we will study how to add interaction information (i.e. DNA methylation interactions, gene interactions, and information which between different diseases) and other biological information to the framework. It would also be interesting to conduct biological experiments to validate our proposed hypotheses for OC-related path associations.

## Supplementary information

A Network-guided Association Mapping Approach from DNA Methylation to Disease

## Data Availability

The software of NAMDD is available at https://github.com/nathanyl/NAMDD.
