# Supplementary material for: A Network-guided Association Mapping Approach from DNA Methylation to Disease
Source: Sci Rep. 2019 Apr 3;9:5601. doi: 10.1038/s41598-019-42010-6 (PMC6447594; doi:10.1038/s41598-019-42010-6)
Supplement: Supplementary file 1 — A Network-guided Association Mapping Approach from DNA Methylation to Disease [file 41598_2019_42010_MOESM1_ESM.pdf]

A Network-guided Association Mapping Approach from DNA Methylation to Disease

——Supplementary Material

Lin Yuan[1] and De-Shuang Huang[1*]

1. Table S1

Number of Samples	Method	AUC	ACC
200	NAMDD (ϕ=0.6)	0.5194	0.5191
	NAMDD (ϕ=0.7)	0.5033	0.5030
	NAMDD (ϕ=0.8)	0.5061	0.5049
	**NsRRR-Logistic**	**0.5704**	**0.5689**
	PCLOGIT	0.5695	0.5689
500	NAMDD (ϕ=0.6)	0.9264	0.8974
	**NAMDD (ϕ=0.7)**	**0.9352**	**0.9010**
	NAMDD (ϕ=0.8)	0.8919	0.8575
	NsRRR-Logistic	0.8181	0.7556
	PCLOGIT	0.6658	0.6214
800	NAMDD (ϕ=0.6)	0.9319	0.9018
	NAMDD (ϕ=0.7)	0.9403	0.9019
	**NAMDD (ϕ=0.8)**	**0.9546**	**0.9137**
	NsRRR-Logistic	0.7505	0.7079
	PCLOGIT	0.6844	0.6215
1100	NAMDD (ϕ=0.6)	0.9279	0.8873
	NAMDD (ϕ=0.7)	0.9600	0.9145
	**NAMDD (ϕ=0.8)**	**0.9717**	**0.9216**
	NsRRR-Logistic	0.8728	0.8247
	PCLOGIT	0.7691	0.7065

Table S1. AUC performances on four simulation datasets for NAMDD with different ϕ and other comparative methods. The AUC values in the table are from Figure 3.

1 Institute of Machine Learning and Systems Biology, College of Electronics and Information Engineering, Tongji University, Shanghai, 201804, P.R. China. Correspondence and requests for materials should be addressed to De-Shuang Huang (email: dshuang@tongji.edu.cn)

2. Table S2

Number of Samples	Method	AUC	ACC
200	NAMDD (ϕ=0.6)	0.5489	0.5142
	NAMDD (ϕ=0.7)	0.5143	0.5089
	NAMDD (ϕ=0.8)	0.5033	0.5012
	**NsRRR-Logistic**	**0.6368**	**0.5872**
	PCLOGIT	0.5852	0.5146
500	NAMDD (ϕ=0.6)	0.9029	0.8547
	**NAMDD (ϕ=0.7)**	**0.9075**	**0.8665**
	NAMDD (ϕ=0.8)	0.9027	0.8471
	NsRRR-Logistic	0.7601	0.7124
	PCLOGIT	0.6216	0.5869
800	NAMDD (ϕ=0.6)	0.9075	0.8575
	NAMDD (ϕ=0.7)	0.9470	0.8964
	**NAMDD (ϕ=0.8)**	**0.9674**	**0.9274**
	NsRRR-Logistic	0.8141	0.7741
	PCLOGIT	0.6479	0.6006
1100	NAMDD (ϕ=0.6)	0.9549	0.9145
	NAMDD (ϕ=0.7)	0.9699	0.9278
	**NAMDD (ϕ=0.8)**	**0.9779**	**0.9305**
	NsRRR-Logistic	0.8755	0.8401
	PCLOGIT	0.7120	0.6558

Table S2. AUC performances on four simulation datasets for NAMDD with different ϕ and other comparative methods. The AUC values in the table are from Figure 4.

3. Table S3

Number of true signatures	NAMDD	NsRRR	PCLOGIT
0	0.00%	0.00%	0.00%
20	0.17%	0.22%	0.27%
40	0.34%	0.39%	0.50%
60	0.53%	0.62%	0.94%
80	0.99%	1.17%	1.25%
100	1.31%	1.63%	2.01%
120	1.45%	2.03%	2.71%
140	1.81%	2.82%	3.24%
160	1.93%	3.72%	4.44%
180	2.37%	4.45%	5.94%
200	2.94%	6.17%	7.20%

Table S3. Detail information of Figure 7.

4. Table S4

DMSs on "mock" dataset	NAMDD	NsRRR	PCLOGIT
0.5%	2.5%	1.0%	0.7%
1.0%	3.6%	1.4%	1.1%
1.5%	5.0%	1.8%	1.8%
2.0%	6.2%	2.5%	2.2%
2.5%	7.6%	3.1%	2.8%
3.0%	9.3%	4.1%	3.6%
3.5%	10.7%	4.4%	3.7%
4.0%	11.7%	5.2%	4.6%
4.5%	12.7%	6.0%	5.1%
5.0%	13.9%	6.6%	5.9%

Table S4. Detail information of Supplementary Figure 1.

5. Table S5

DNA methylation site	Gene	PCC	P-value
cg20822628	*CCNE1*	-0.4387	7.22e-29
cg00499822	*CCNE1*	-0.4484	3.10e-30
cg23588759	*CCNE1*	-0.3459	7.35e-18
cg03504078	*CCNE1*	-0.3602	2.49e-19
cg08861115	*CCNE1*	-0.4750	3.33e-34
cg21906716	*CCNE1*	-0.3544	9.99e-19
cg19290962	*CCNE1*	-0.3667	4.99e-20
cg16935609	*CCNE1*	-0.3737	8.66e-21
cg09111917	*CCNE1*	-0.3941	3.82e-23
cg01309153	*CCNE1*	-0.5558	1.18e-48
cg16391792	*AURKA*	-0.3928	5.47e-23
cg05697231	*AURKA*	-0.3485	4.07e-18
cg19515446	*AURKA*	-0.4807	4.22e-35
cg19035993	*AURKA*	-0.4469	5.15e-30
cg04711324	*AURKA*	-0.3848	4.70e-22
cg17675150	*AURKA*	-0.4769	3.50e-34
cg19320612	*AURKA*	-0.3780	2.80e-21
cg22313025	*RAB25*	-0.4062	1.29e-24
cg23303782	*RAB25*	-0.5476	5.31e-47
cg17749520	*RAB25*	-0.3890	1.54e-22
cg08377000	*RAB25*	-0.5555	1.39e-48
cg03381111	*RAB25*	-0.4707	1.54e-33

Table S5. Pearson correlation coefficients (PCCs) of DNA methylation sites and genes in disease samples from 22 paths.

6. Table S6

DNA methylation site	Start position	Gene	Chromosome	Start position-End position	NES	NOM p-val	FDR q-val
cg20822628	chr20:61041592	*GATA5*	chr20	61038552-61051026	-2.10638	7.23E-06	4.81E-05
cg21906716	chr1: 3579978	*TP73*	chr1	3569128-3652765	-2.01245	8.93E-05	1.64E-04
cg05697231	chr15:74286614	*PML*	chr15	74287013-74340155	-2.0313	6.63E-05	1.28E-04
cg19515446	chr6:26108335	*HIST1H1T*	chr6	26107639-26108364	-2.05297	1.14E-06	8.75E-05
cg17675150	chr18:56529784	*ZNF532*	chr18	56529831-56653712	-2.06345	9.34E-07	4.58E-05
cg08377000	chr4:90033921	*TIGD2*	chr4	90033967-90036052	-2.05522	3.22E-06	8.96E-05

Table S6. Detailed location information of 6 DNA methylation sites and nearby transcription factor genes.

7. Table S7

DNA methylation site	Gene	PCC	P-value	Annotation
cg20822628	*CCNE1*	-0.4387	7.22E-29	gene body
cg00499822	*CCNE1*	-0.4484	3.10E-30	gene body
cg23588759	*CCNE1*	-0.3459	7.35E-18	gene body
cg03504078	*CCNE1*	-0.3602	2.49E-19	Intergenic
cg08861115	*CCNE1*	-0.4750	3.33E-34	gene body
cg21906716	*CCNE1*	-0.3544	9.99E-19	gene body
cg19290962	*CCNE1*	-0.3667	4.99E-20	promoter
cg16935609	*CCNE1*	-0.3737	8.66e-21	promoter
cg09111917	*CCNE1*	-0.3941	3.82E-23	promoter
cg01309153	*CCNE1*	-0.5558	1.18E-48	Intergenic
cg16391792	*AURKA*	-0.3928	5.47E-23	gene body
cg05697231	*AURKA*	-0.3485	4.07E-18	promoter
cg19515446	*AURKA*	-0.4807	4.22E-35	gene body
cg19035993	*AURKA*	-0.4469	5.15E-30	gene body
cg04711324	*AURKA*	-0.3848	4.70E-22	gene body
cg17675150	*AURKA*	-0.4769	3.50E-34	promoter
cg19320612	*AURKA*	-0.3780	2.80E-21	gene body
cg22313025	*RAB25*	-0.4062	1.29E-24	gene body
cg23303782	*RAB25*	-0.5476	5.31E-47	gene body
cg17749520	*RAB25*	-0.3890	1.54E-22	gene body
cg18808261	*RFC4*	-0.3332	8.05E-29	promoter
cg03724463	*RFC4*	-0.2901	7.93E-06	Intergenic
cg27462398	*RFC4*	-0.4948	7.57E-34	Intergenic
cg00515905	*RFC4*	-0.3761	9.67E-05	gene body
cg27413508	*TPX2*	-0.4164	4.89E-26	promoter
cg09027725	*TPX2*	0.2459	7.81E-23	promoter

cg14701962	*TPX2*	0.1959	4.38E-29	gene body
cg08623787	*TPX2*	-0.2677	5.08E-24	gene body
cg01040850	*TPX2*	-0.1022	8.77E-08	promoter
cg00826384	*TPX2*	-0.2923	3.67E-23	Intergenic
cg07550362	*ASNS*	-0.3799	3.99E-44	promoter
cg14221171	*ASNS*	0.1769	5.79E-17	promoter
cg21428681	*ASNS*	0.1339	5.55E-17	gene body
cg20910746	*ASNS*	-0.1274	3.56E-42	Intergenic
cg04463638	*CLDN5*	0.1536	2.74E-24	promoter
cg11450827	*CLDN5*	-0.2560	4.63E-12	promoter
cg21274025	*CLDN5*	0.5564	2.98E-14	Intergenic
cg24356544	*CLDN5*	-0.1293	8.37E-33	gene body
cg17030820	*CLDN5*	-0.3266	3.36E-35	Intergenic
cg19740375	*CLDN5*	-0.1321	4.15E-17	gene body
cg02254461	*CLDN5*	0.4434	8.56E-20	Intergenic
cg08860143	*CLDN5*	-0.3295	6.75E-45	gene body
cg01344171	*VEGFA*	-0.4477	8.96E-46	promoter
cg04600618	*VEGFA*	-0.3148	4.40E-37	promoter
cg22407458	*VEGFA*	0.4657	8.49E-22	Intergenic
cg01108476	*VEGFA*	0.2598	9.75E-12	gene body
cg21604042	*VEGFA*	0.4507	6.04E-16	promoter
cg00347904	*VEGFA*	-0.3666	9.74E-50	Intergenic
cg06656924	*MUC1*	-0.4407	6.05E-22	promoter
cg25994725	*MUC1*	-0.3310	9.69E-33	gene body
cg25107791	*MUC1*	0.3362	9.17E-09	promoter
cg04058169	*MUC1*	-0.4966	2.07E-27	gene body
cg01985396	*MUC1*	-0.3696	4.98E-46	gene body
cg07403350	*MUC1*	-0.2137	9.51E-46	gene body
cg13468685	*MUC1*	0.4518	7.71E-15	gene body
cg18221862	*LPHN2*	0.2142	2.71E-24	promoter
cg25870263	*LPHN2*	0.4246	7.74E-05	gene body
cg16812893	*LPHN2*	-0.2889	9.72E-23	gene body
cg11158374	*LPHN2*	0.1253	5.83E-39	Intergenic
cg11752275	*LPHN2*	-0.6267	7.64E-41	promoter
cg02873524	*VPS39*	-0.3800	3.10E-25	gene body
cg22398616	*VPS39*	0.1041	9.34E-06	promoter
cg19404190	*VPS39*	0.4158	4.87E-30	gene body
cg13883681	*LGALS8*	-0.3175	2.78E-22	promoter
cg24400943	*LGALS8*	-0.4702	1.31E-28	gene body
cg22836229	*LGALS8*	-0.5304	9.60E-24	promoter
cg11976616	*ARTS-1*	0.4871	6.89E-45	Intergenic
cg08368934	*ARTS-1*	-0.3683	6.63E-10	promoter
cg19876838	*ARTS-1*	-0.2418	7.56E-14	Intergenic

cg07703401	*POU4F1*	-0.3735	7.74E-43	promoter
cg07897701	*POU4F1*	-0.2614	7.00E-31	promoter
cg1956296	*POU4F1*	-0.1368	7.74E-27	gene body
cg13975625	*POU4F1*	0.2202	8.77E-22	promoter
cg01530101	*POU4F1*	0.2276	4.58E-28	Intergenic
cg09076077	*GPR161*	-0.4795	7.25E-37	gene body
cg06498267	*GPR161*	-0.3241	5.65E-10	promoter
cg05396987	*GPR161*	-0.1129	7.35E-15	promoter
cg25157408	*MGST3*	-0.2683	1.29E-29	gene body
cg07837085	*MGST3*	-0.2418	4.53E-05	promoter
cg01684579	*MGST3*	-0.2735	9.61E-33	promoter
cg16612699	*MGST3*	-0.2691	4.54E-42	promoter
cg02712878	*MGST3*	-0.1377	9.51E-39	promoter
cg20053158	*TRHR*	-0.3394	8.60E-32	promoter
cg03001305	*TRHR*	-0.2884	4.12E-25	Intergenic
cg18396533	*TRHR*	-0.3784	1.60E-48	gene body
cg19576304	*TRHR*	0.1422	4.46E-29	Intergenic
cg16107172	*SAC3D1*	-0.3444	6.33E-11	gene body
cg16001913	*SAC3D1*	-0.4115	7.30E-38	promoter
cg23973000	*SAC3D1*	0.2694	9.92E-22	gene body
cg21432954	*SH3BGRL3*	-0.1363	4.66E-09	gene body
cg16636110	*SH3BGRL3*	-0.2066	1.76E-06	promoter
cg16248277	*SH3BGRL3*	-0.1614	5.84E-17	gene body
cg02066681	*SH3BGRL3*	0.2124	4.46E-29	promoter
cg00633969	*FXR2*	-0.2760	6.33E-11	gene body
cg20022122	*FXR2*	-0.3109	7.30E-38	promoter
cg21233722	*FXR2*	-0.2830	9.92E-22	gene body
cg10157098	*KLHL20*	-0.4502	2.96E-12	promoter
cg05847038	*KLHL20*	-0.3072	3.42E-35	gene body
cg05159188	*KLHL20*	-0.4775	3.90E-29	gene body
cg05711886	*KLHL20*	0.3551	7.07E-20	promoter
cg23534142	*KLHL20*	-0.4831	6.82E-18	gene body
cg01476044	*KLHL20*	-0.1963	5.09E-15	gene body
cg19721889	*NR1D1*	-0.3705	6.91E-48	promoter
cg26385286	*NR1D1*	-0.2156	4.12E-28	Intergenic
cg02983451	*NR1D1*	-0.3687	6.03E-04	gene body
cg13755070	*NR1D1*	-0.3781	8.06E-07	gene body
cg20300655	*NR1D1*	-0.1272	7.44E-12	Intergenic
cg16222568	*NR1D1*	-0.2019	7.04E-43	gene body
cg06063714	*UBXD1*	-0.1896	6.40E-12	gene body
cg11248413	*UBXD1*	-0.3671	7.09E-13	Intergenic
cg18546622	*UBXD1*	-0.4378	6.02E-12	gene body
cg05358291	*UBXD1*	-0.2378	4.38E-45	gene body

cg23749046	*UBXD1*	-0.4122	6.75E-47	gene body
cg16742703	*CDK2AP1*	-0.3702	5.47E-39	gene body
cg01124420	*CDK2AP1*	-0.1026	9.92E-39	gene body
cg16352283	*CDK2AP1*	-0.3409	8.54E-11	promoter
cg02149446	*CDK2AP1*	-0.2547	7.82E-22	Intergenic
cg04041960	*CDK2AP1*	-0.4664	6.92E-46	promoter
cg18788940	*KLF7*	-0.1004	5.13E-25	gene body
cg04016326	*KLF7*	-0.2850	6.66E-41	promoter
cg13885201	*KLF7*	-0.2697	9.78E-05	gene body
cg19646028	*KLF7*	-0.2918	1.37E-09	promoter
cg25300386	*KLF7*	-0.3557	5.29E-33	gene body
cg13626881	*PDE4B*	-0.3179	9.81E-29	promoter
cg12391048	*PDE4B*	-0.3589	6.58E-42	gene body
cg06403553	*PDE4B*	-0.3176	2.24E-26	promoter
cg20837735	*BLZF1*	-0.3884	8.05E-45	gene body
cg00546491	*BLZF1*	-0.3090	7.50E-22	gene body
cg04376617	*BLZF1*	-0.2844	6.01E-11	gene body
cg03580247	*HK3*	-0.4081	7.90E-43	gene body
cg23257840	*HK3*	0.2290	4.54E-04	gene body
cg23627134	*HK3*	-0.4139	4.02E-12	gene body
cg20322977	*HK3*	-0.2885	9.74E-06	gene body
cg16722118	*OSGIN1*	-0.1143	1.81E-15	gene body
cg16864658	*OSGIN1*	-0.1703	3.86E-10	promoter
cg05671350	*OSGIN1*	-0.3887	5.48E-28	gene body
cg25677688	*BHMT2*	-0.2894	8.06E-25	gene body
cg06785429	*BHMT2*	-0.1611	2.45E-37	promoter
cg16698623	*BHMT2*	-0.2364	7.68E-43	gene body
cg00556408	*BHMT2*	-0.343	9.82E-15	promoter
cg02876062	*BHMT2*	-0.1767	5.51E-50	gene body
cg08933517	*BHMT2*	-0.3954	4.86E-48	promoter
cg24378421	*OR1G1*	-0.1971	9.95E-34	promoter
cg21438018	*OR1G1*	-0.467	5.96E-41	Intergenic
cg22445920	*OR1G1*	-0.2076	7.22E-20	Intergenic
cg13818573	*STXBP1*	-0.4062	3.93E-49	promoter
cg26656135	*STXBP1*	-0.1754	6.54E-48	promoter
cg09009380	*STXBP1*	-0.215	3.12E-12	promoter
cg16415058	*NPL*	-0.1364	3.97E-33	Intergenic
cg12614105	*NPL*	-0.3305	1.17E-07	gene body
cg19378133	*NPL*	-0.3734	4.26E-41	gene body
cg03609102	*NPL*	-0.3186	9.22E-30	gene body
cg09911755	*NPL*	-0.2703	8.48E-10	promoter
cg22081096	*TRMU*	-0.3578	6.05E-36	gene body
cg16990174	*TRMU*	-0.3591	9.25E-50	gene body

cg11108890	*TRMU*	-0.3716	2.98E-04	promoter
cg17040807	*TRMU*	-0.3543	3.89E-22	gene body
cg01335367	*TRMU*	-0.4781	5.05E-31	gene body
cg14155416	*CACNA2D3*	-0.1835	8.72E-46	promoter
cg27390220	*CACNA2D3*	-0.3837	7.04E-30	gene body
cg10885338	*CACNA2D3*	-0.1945	9.94E-44	promoter
cg03782727	*PRKCSH*	-0.1477	8.32E-46	gene body
cg16773028	*PRKCSH*	-0.3429	6.19E-24	promoter
cg20895877	*PRKCSH*	-0.2801	2.40E-25	gene body
cg05483509	*PRKCSH*	-0.1254	5.75E-06	promoter
cg26756083	*PRKCSH*	-0.2618	4.19E-06	gene body
cg05902852	*PRKCSH*	-0.2793	6.15E-43	promoter
cg14785479	*ARSB*	0.2463	1.97E-05	promoter
cg24469977	*ARSB*	-0.4054	7.30E-44	gene body
cg15042080	*ARSB*	-0.3512	9.52E-28	promoter
cg10523019	*ARSB*	-0.4088	4.28E-18	gene body
cg14948436	*ARSB*	-0.2835	4.19E-23	gene body
cg26170660	*ARSB*	-0.3648	8.46E-40	gene body
cg16855440	*ATM*	-0.4081	4.24E-39	promoter
cg25370441	*ATM*	0.2401	9.77E-34	Intergenic
cg03835296	*ATM*	-0.3648	8.02E-35	gene body
cg26738880	*C8orf17*	-0.2665	6.38E-10	promoter
cg24125648	*C8orf17*	-0.4368	1.54E-07	gene body
cg00767581	*C8orf17*	-0.4332	4.83E-43	gene body
cg09424896	*ERCC3*	-0.2026	5.54E-16	promoter
cg17983307	*ERCC3*	-0.3454	2.70E-10	gene body
cg16386080	*ERCC3*	-0.3329	5.93E-06	promoter
cg06836772	*ERCC3*	-0.3163	7.17E-37	promoter
cg20427865	*IKBKB*	-0.448	7.29E-38	gene body
cg26033681	*IKBKB*	-0.2059	2.52E-10	promoter
cg22356117	*IKBKB*	-0.2272	9.66E-35	Intergenic
cg25922353	*UPF3B*	-0.1476	4.86E-19	promoter
cg10414946	*UPF3B*	-0.476	8.95E-22	gene body
cg27022827	*UPF3B*	-0.3582	6.56E-47	promoter
cg07221454	*UPF3B*	0.1177	7.81E-32	gene body
cg04201347	*UPF3B*	-0.4020	2.92E-22	Intergenic
cg14015044	*UPF3B*	-0.1971	3.48E-33	promoter
cg05089968	*ELF2*	-0.2770	6.56E-30	gene body
cg24428042	*ELF2*	0.3751	1.46E-14	promoter
cg00393585	*ELF2*	-0.2437	9.58E-07	gene body
cg03387497	*PHOX2A*	-0.3946	2.08E-47	gene body
cg26981881	*PHOX2A*	-0.2579	7.08E-50	promoter
cg14401592	*PHOX2A*	0.3734	2.64E-08	gene body

cg02982690	*PHOX2A*	-0.3816	7.25E-49	Intergenic
cg22797991	*PHOX2A*	-0.2769	2.09E-16	gene body
cg18971054	*CD3G*	0.1078	6.03E-18	gene body
cg08687163	*CD3G*	-0.2323	2.85E-46	promoter
cg04993257	*CD3G*	-0.2697	3.71E-04	promoter
cg14839898	*CD3G*	-0.2081	5.08E-22	promoter
cg10293925	*CD3G*	-0.1788	6.27E-39	promoter
cg17952262	*CD3G*	0.4287	2.24E-32	promoter
cg05820087	*SOX3*	-0.2720	3.17E-15	gene body
cg07908874	*SOX3*	-0.4551	4.36E-31	gene body
cg02283643	*SOX3*	-0.2565	8.29E-22	Intergenic
cg17880199	*SOX3*	-0.4077	5.96E-05	gene body
cg27433062	*SOX3*	0.2587	5.31E-14	Intergenic
cg04601137	*SOX3*	-0.4234	7.31E-21	gene body
cg20764656	*TIMM8A*	0.4021	6.61E-19	promoter
cg21375825	*TIMM8A*	-0.2509	6.24E-31	gene body
cg02085507	*TIMM8A*	-0.3058	9.85E-48	gene body
cg07084746	*TIMM8A*	-0.4538	9.41E-09	promoter
cg13302154	*TIMM8A*	-0.3352	4.72E-27	promoter
cg22587758	*TIMM8A*	-0.1619	3.08E-22	gene body
cg04856858	*EIF3G*	0.1799	2.20E-05	promoter
cg17657618	*EIF3G*	-0.2628	9.09E-25	gene body
cg27622610	*EIF3G*	-0.3995	2.47E-19	Intergenic
cg08977371	*EIF3G*	0.4303	7.58E-20	gene body
cg02154186	*EIF3G*	-0.4160	1.90E-27	promoter
cg05440289	*RTCD1*	-0.2274	3.41E-12	gene body
cg10193817	*RTCD1*	-0.3136	5.97E-16	promoter
cg26523005	*RTCD1*	0.1359	6.85E-38	promoter
cg07080946	*RTCD1*	-0.1446	3.06E-31	gene body
cg12493906	*RTCD1*	-0.1545	5.28E-46	promoter
cg01593385	*SFXN1*	0.3715	4.63E-47	promoter
cg14849423	*SFXN1*	0.2981	6.36E-46	gene body
cg10922280	*SFXN1*	-0.1759	2.61E-33	Intergenic
cg07173760	*SFXN1*	-0.2980	8.51E-46	gene body
cg11721194	*SFXN1*	0.1590	8.71E-50	promoter
cg04555771	*TBRG4*	-0.1219	4.00E-14	promoter
cg26705553	*TBRG4*	-0.4403	6.29E-49	gene body
cg16131766	*TBRG4*	0.3242	9.65E-39	gene body
cg03760483	*TBRG4*	-0.4719	5.13E-49	promoter
cg01558777	*RRP1*	-0.3787	2.86E-40	gene body
cg11251498	*RRP1*	-0.3331	2.21E-19	gene body
cg20784775	*RRP1*	0.4262	9.36E-23	gene body
cg16118817	*RRP1*	0.4516	9.42E-36	gene body

cg00951770	*ME3*	-0.4956	1.77E-27	gene body
cg22289115	*ME3*	-0.1002	6.96E-28	promoter
cg17307280	*ME3*	-0.4462	8.11E-25	Intergenic
cg16992787	*ME3*	-0.3450	9.22E-40	gene body
cg12970084	*SLC22A18AS*	-0.4960	9.07E-27	gene body
cg21755709	*SLC22A18AS*	0.3111	9.36E-23	gene body
cg20876010	*SLC22A18AS*	-0.2918	9.42E-36	Intergenic
cg04272086	*PRKG1*	-0.4206	1.77E-27	gene body
cg25602457	*PRKG1*	-0.1911	6.96E-28	gene body
cg08361238	*PRKG1*	-0.2992	8.11E-25	promoter
cg03506489	*GTF2H4*	0.4604	7.90E-19	gene body
cg17692403	*GTF2H4*	0.3299	5.40E-13	promoter
cg22282941	*GTF2H4*	-0.4381	3.94E-43	gene body
cg24456340	*GTF2H4*	-0.3955	3.32E-19	gene body
cg04956511	*GTF2H4*	-0.3344	9.45E-24	gene body
cg01636591	*ADAMTS5*	0.1987	7.86E-45	gene body
cg15787039	*ADAMTS5*	-0.3666	5.85E-08	gene body
cg23239396	*ADAMTS5*	0.1334	7.97E-38	Intergenic
cg00350296	*ADAMTS5*	-0.3504	1.74E-35	gene body
cg09381003	*ADAMTS5*	-0.3644	9.10E-48	gene body
cg00662556	*SFRS8*	-0.3919	2.38E-48	promoter
cg19025034	*SFRS8*	0.4563	2.04E-06	promoter
cg08351331	*SFRS8*	0.4930	9.89E-08	gene body
cg19058765	*SFRS8*	-0.4076	1.48E-41	promoter
cg21906716	*GPR68*	-0.3326	4.56E-37	promoter
cg08840010	*GPR68*	-0.4714	7.61E-48	gene body
cg07713493	*GPR68*	0.3320	8.22E-50	promoter
cg26504021	*KRT5*	-0.1067	8.69E-05	Intergenic
cg21488617	*KRT5*	0.1483	1.00E-29	gene body
cg25738273	*KRT5*	-0.4451	9.65E-24	promoter
cg20011134	*KRT5*	-0.2937	5.69E-08	promoter
cg11981631	*KRT5*	-0.4380	3.33E-49	gene body
cg11004890	*KRT5*	-0.1837	4.00E-06	Intergenic
cg03032025	*MARCO*	0.3209	3.65E-11	gene body
cg08001895	*MARCO*	0.3520	6.42E-39	promoter
cg12387247	*MARCO*	-0.1127	2.61E-19	promoter
cg01500097	*MARCO*	-0.3459	3.07E-37	promoter
cg27420236	*MARCO*	-0.2450	4.59E-06	promoter
cg15457079	*MARCO*	-0.1198	5.94E-38	gene body
cg14333565	*GLE1L*	0.2958	7.45E-17	gene body
cg10281478	*GLE1L*	-0.1770	5.98E-28	promoter
cg22090592	*GLE1L*	-0.1492	4.50E-31	Intergenic
cg06317209	*CCNA1*	-0.1822	6.90E-25	gene body

cg19252956	*CCNA1*	-0.1586	4.92E-12	promoter
cg01027739	*CCNA1*	0.1756	1.01E-18	promoter
cg09260441	*HLA-DPB1*	0.1170	4.07E-47	gene body
cg21602160	*HLA-DPB1*	-0.3541	8.46E-44	promoter
cg22427279	*HLA-DPB1*	-0.2127	8.84E-19	promoter
cg14958635	*HLA-DPB1*	-0.3154	2.04E-06	Intergenic
cg23101680	*PARP16*	-0.3781	5.17E-04	promoter
cg05755354	*PARP16*	0.2996	4.66E-45	promoter
cg06812844	*PARP16*	0.3143	6.45E-15	promoter
cg02164442	*PARP16*	-0.2781	5.88E-19	Intergenic
cg08012287	*PARP16*	-0.1495	7.86E-42	promoter
cg19885761	*PARP16*	-0.2961	3.34E-24	gene body
cg01324261	*RNF41*	-0.4412	3.97E-14	gene body
cg02197293	*RNF41*	-0.4496	4.31E-48	promoter
cg09120035	*RNF41*	0.2081	8.39E-30	Intergenic
cg24459563	*RNF41*	-0.1834	8.62E-15	gene body
cg18330203	*RNF41*	-0.4792	3.65E-35	gene body
cg26465611	*KCNH1*	-0.1328	1.39E-30	Intergenic
cg12188860	*KCNH1*	-0.1422	1.28E-20	gene body
cg07376232	*KCNH1*	0.1568	2.09E-45	gene body
cg06546607	*KCNH1*	-0.1666	9.06E-32	promoter
cg24794433	*CLSPN*	-0.3484	6.97E-09	gene body
cg26323655	*CLSPN*	0.3295	8.73E-49	Intergenic
cg22784047	*CLSPN*	-0.1208	9.50E-40	gene body
cg21152671	*NT5C*	0.4725	9.44E-31	promoter
cg05250458	*NT5C*	-0.3915	4.38E-28	promoter
cg09201327	*NT5C*	-0.3952	7.55E-12	gene body
cg11639651	*NT5C*	-0.1253	6.35E-49	promoter
cg20587968	*NT5C*	-0.4442	1.54E-38	gene body
cg10189695	*PTPRN2*	0.4738	8.10E-21	promoter
cg12022621	*PTPRN2*	-0.4938	9.95E-49	gene body
cg10544564	*PTPRN2*	0.4436	3.79E-07	gene body
cg19756068	*SPOCK3*	-0.4143	3.09E-05	gene body
cg27558666	*SPOCK3*	-0.3054	1.32E-39	gene body
cg12819826	*SPOCK3*	-0.1710	3.93E-08	promoter
cg01733599	*CRLF1*	-0.2594	6.49E-41	promoter
cg13899108	*CRLF1*	-0.1535	4.95E-40	Intergenic
cg08013810	*CRLF1*	0.1123	7.26E-28	promoter
cg20959523	*CRLF1*	-0.4757	2.79E-24	gene body
cg04106190	*CRLF1*	-0.2205	3.43E-14	Intergenic
cg08088989	*CRLF1*	-0.2182	5.75E-05	promoter
cg00910168	*DOLK*	-0.2332	6.25E-29	gene body
cg21621114	*DOLK*	-0.2868	2.89E-27	gene body

cg06711560	*DOLK*	-0.3593	6.62E-31	promoter
cg06388544	*OTC*	-0.1100	4.08E-38	promoter
cg26153631	*OTC*	-0.4369	6.19E-05	promoter
cg03802191	*OTC*	-0.3236	9.06E-42	Intergenic
cg01618851	*OTC*	0.4417	3.97E-24	gene body
cg19987219	*OTC*	-0.2391	5.13E-06	gene body
cg27256309	*PLOD3*	-0.2784	8.55E-29	promoter
cg26267310	*PLOD3*	0.1217	1.23E-48	gene body
cg22009923	*PLOD3*	0.1708	7.29E-33	promoter
cg13481359	*PLOD3*	-0.4589	4.51E-16	gene body
cg04111761	*PLOD3*	-0.1786	8.10E-18	promoter
cg08996748	*PLOD3*	0.1373	6.85E-49	gene body
cg04180953	*PDXK*	-0.2229	3.73E-31	promoter
cg22035229	*PDXK*	-0.2824	9.23E-34	gene body
cg13782134	*PDXK*	0.1406	5.93E-21	promoter
cg07675169'	*PDXK*	0.4982	3.91E-22	promoter
cg27196467	*PDXK*	-0.3651	5.75E-23	promoter
cg11716026	*PDXK*	0.2323	7.95E-18	gene body
cg05985767	*ZNF787*	-0.4594	6.96E-17	promoter
cg03028472	*ZNF787*	-0.1472	1.84E-47	gene body
cg11286122	*ZNF787*	-0.4954	8.95E-48	promoter
cg10457247	*ZNF787*	-0.3160	4.66E-16	gene body
cg22416721	*ZNF787*	-0.3828	6.13E-50	promoter
cg26021627	*ZNF787*	-0.4998	7.35E-09	promoter
cg25995460	*LOXL1*	-0.2151	4.96E-48	gene body
cg15583072	*LOXL1*	-0.2658	3.38E-07	promoter
cg03330516	*LOXL1*	-0.2859	4.24E-12	gene body
cg19109050	*LOXL1*	0.4056	2.67E-13	promoter
cg25604883	*LOXL1*	-0.4273	6.00E-28	gene body
cg23022999	*LOXL1*	-0.1400	5.79E-37	promoter
cg08924430	*ARPC5L*	-0.1712	5.81E-49	gene body
cg11037787	*ARPC5L*	-0.2438	2.63E-08	promoter
cg10275770	*ARPC5L*	-0.1226	6.46E-09	gene body
cg15739581	*ARPC5L*	0.3088	2.39E-10	gene body
cg15743985	*ARPC5L*	-0.2343	7.72E-39	promoter
cg19789505	*OSBP*	-0.1702	3.09E-47	Intergenic
cg11251858	*OSBP*	-0.3651	2.33E-33	gene body
cg10377274	*OSBP*	-0.2323	1.53E-45	promoter
cg25101936	*OSBP*	0.4594	2.51E-39	promoter
cg05877109	*EPB42*	0.1472	3.88E-42	gene body
cg07126559	*EPB42*	-0.4954	8.44E-19	Intergenic
cg04845579	*EPB42*	-0.3160	6.25E-28	promoter
cg06564900	*EPB42*	-0.3828	9.15E-33	gene body

cg01015871	*EPB42*	-0.4998	6.94E-06	gene body
cg08046471	*EPB42*	-0.2151	8.15E-29	promoter
cg24454143	*MLLT4*	-0.2658	5.01E-34	gene body
cg03003256	*MLLT4*	-0.2859	4.51E-27	promoter
cg00278366	*MLLT4*	-0.4056	5.42E-07	gene body
cg11191210	*MLLT4*	-0.4273	3.05E-43	gene body
cg07693270	*MLLT4*	-0.1400	5.98E-35	gene body
cg09076584	*MLLT4*	-0.1712	4.87E-49	gene body
cg18952647	*BLVRA*	-0.2438	5.88E-24	promoter
cg25044651	*BLVRA*	0.1226	2.36E-22	Intergenic
cg12237269	*BLVRA*	0.3088	5.06E-39	promoter
cg23290344	*BLVRA*	-0.1835	1.68E-31	gene body
cg03455458	*BLVRA*	-0.4621	5.77E-46	promoter
cg17791651	*BLVRA*	-0.3702	1.01E-05	promoter
cg11673092	*TCN1*	-0.2874	2.87E-24	Intergenic
cg12880658	*TCN1*	0.4649	4.65E-22	Intergenic
cg07476030	*TCN1*	0.1416	4.37E-10	gene body
cg10757144	*TCN1*	-0.3982	1.98E-09	promoter
cg26113512	*TCN1*	-0.3945	4.72E-18	gene body

Table S7. PCCs and P-values between methylation sites and genes.

8. Figure S1

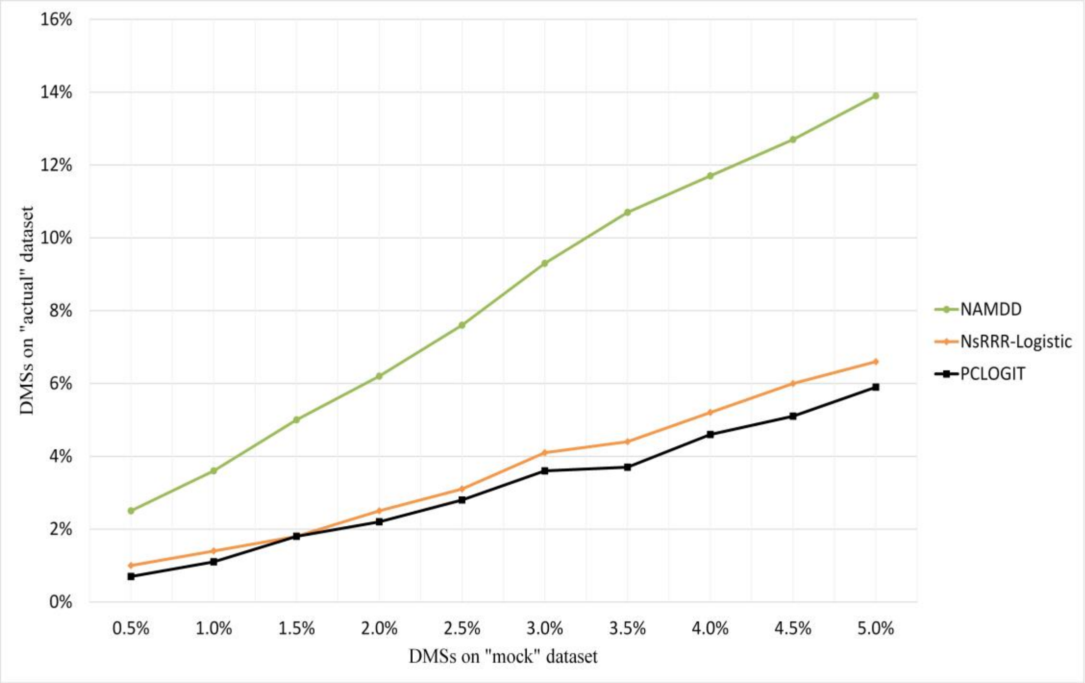

Figure S1. Comparison of differential algorithms on real ovarian cancer dataset experiment with sample-exchange test. NAMDD outperforms the competing methods.

9. More hypothetical path associations

In the path association cg05697231→AURKA→OC, cg05697231 (chr15: 73994273-73994274) is located 445 bp upstream of the TSS of PML (promyelocytic leukemia). PML is one of the most important tumor suppressors[1,2]. It has been reported that PML directly interacts with AURKA during G1, S, and G2/M phase of the cell cycle[3]. Based on the above mentioned information, we hypothesize the biological regulation mechanism as follows. First, the cg05697231 site results in silencing or down-regulation of the PML. Second, the perturbed PML level causes overexpression of AURKA. Finally, the high level of AURKA promotes ovarian cancer cell proliferation. The discovery of Tsou et al.[4] is similar to the hypothesis and provides evidence for our hypothesis. In the future work, it would be interesting to research the cg05697231-PML-AURKA-OC pathway through biological experiment.

In the path association cg23303782→RAB25→OC, cg23303782 (chr10: 119208232-119208233) is located 642 bp downstream of the TSS of GRK5 (g protein-coupled receptor kinase 5). GRK5 encodes a member of the guanine nucleotide-binding protein (G protein)-coupled receptor kinase (GRK) subfamily of the Ser/Thr protein kinase family[5]. G protein activates cAMP (cyclic adenosine monophosphate)-dependent protein kinases through signaling pathways. It has been reported that cAMP regulates the expression of RAB25 through cAMP-PKA (protein kinase A)-CREB (cAMP-response element binding protein) pathway[6]. Combining the above mentioned information, we hypothesize the biological regulation mechanism as follows. First, the cg23303782 site affects the expression level of GRK5 and leads the high expression level of cAMP. Second, the cAMP activates the pathway cAMP-PKA-CREB which leads to the overexpression of RAB25. Finally, the overexpression of RAB25 increases the possibility of cancer cell proliferation and survival.

References

1 Valdez, K. E. *et al.* NEMO, a transcriptional target of estrogen and progesterone, is linked to tumor suppressor PML in breast cancer. *Cancer Research* **77** (2017).

2 Trotman, L. C. *et al.* Identification of a tumour suppressor network opposing nuclear Akt function. *Nature* **441**, 523-527 (2006).

3 Dephoure, N. *et al.* A quantitative atlas of mitotic phosphorylation. *Proceedings of the National Academy of Sciences of the United States of America* **105**, 10762-10767 (2008).

4 Tsou, J. H. *et al.* Aberrantly expressed AURKC enhances the transformation and tumourigenicity of epithelial cells. *Journal of Pathology* **225**, 243–254 (2011).

5 Liggett, S. B. *et al.* A GRK5 Polymorphism that Inhibits β-Adrenergic Receptor Signaling is Protective in Heart Failure. *Nature Medicine* **14**, 510 (2008).

6 Xue, H. *et al.* A CRE that binds CREB and contributes to PKA-dependent regulation of the proximal promoter of human RAB25 gene. *International Journal of Biochemistry & Cell Biology* **43**, 348-357 (2011).
